# Multi-label transcriptional classification of colorectal cancer reflects tumor cell population heterogeneity

**DOI:** 10.1186/s13073-023-01176-5

**Published:** 2023-05-15

**Authors:** Silvia Cascianelli, Chiara Barbera, Alexandra Ambra Ulla, Elena Grassi, Barbara Lupo, Diego Pasini, Andrea Bertotti, Livio Trusolino, Enzo Medico, Claudio Isella, Marco Masseroli

**Affiliations:** 1 Department of Electronics, Information and Bioengineering, Politecnico Di Milano, Piazza Leonardo da Vinci 32, 20133 Milan, Italy; 2 Department of Oncology, University of Turin, S.P. 142, Km 3.95, 10060 Candiolo (TO), Turin, Italy; 3 Candiolo Cancer Institute, FPO-IRCCS, S.P. 142, Km 3.95, 10060 Candiolo (TO), Italy; 4 Department of Experimental Oncology, IEO, European Institute of Oncology IRCCS, Via Adamello 16, 20139 Milan, Italy; 5 Department of Health Sciences, University of Milan, Via A. Di Rudini 8, 20142 Milan, Italy

**Keywords:** Colorectal cancer, Molecular subtyping, Computational biology, Tumor heterogeneity

## Abstract

**Background:**

Transcriptional classification has been used to stratify colorectal cancer (CRC) into molecular subtypes with distinct biological and clinical features. However, it is not clear whether such subtypes represent discrete, mutually exclusive entities or molecular/phenotypic states with potential overlap. Therefore, we focused on the CRC Intrinsic Subtype (CRIS) classifier and evaluated whether assigning multiple CRIS subtypes to the same sample provides additional clinically and biologically relevant information.

**Methods:**

A multi-label version of the CRIS classifier (multiCRIS) was applied to newly generated RNA-seq profiles from 606 CRC patient-derived xenografts (PDXs), together with human CRC bulk and single-cell RNA-seq datasets. Biological and clinical associations of single- and multi-label CRIS were compared. Finally, a machine learning-based multi-label CRIS predictor (ML^2^CRIS) was developed for single-sample classification.

**Results:**

Surprisingly, about half of the CRC cases could be significantly assigned to more than one CRIS subtype. Single-cell RNA-seq analysis revealed that multiple CRIS membership can be a consequence of the concomitant presence of cells of different CRIS class or, less frequently, of cells with hybrid phenotype. Multi-label assignments were found to improve prediction of CRC prognosis and response to treatment. Finally, the ML^2^CRIS classifier was validated for retaining the same biological and clinical associations also in the context of single-sample classification.

**Conclusions:**

These results show that CRIS subtypes retain their biological and clinical features even when concomitantly assigned to the same CRC sample. This approach could be potentially extended to other cancer types and classification systems.

**Supplementary Information:**

The online version contains supplementary material available at 10.1186/s13073-023-01176-5.

## Background

Despite the advancements in translational oncology, colorectal carcinoma (CRC) is still the second leading cause of cancer death over the world [1]. To improve therapy decision making, in the last decades several transcriptome-based tumor classifiers have been developed to stratify patients into groups with unique molecular, biological, and clinical hallmarks [2–7]. To streamline clinical practice, in 2015 the CRC Subtyping Consortium (CRCSC) integrated independent classification criteria to obtain four Consensus Molecular Subtypes (CMS) [8]. This classification was obtained from the gene expression of bulk cancer tissues strongly admixed with by transcripts of stromal origin, as later revealed [9, 10], so that the CMS4 subtype is highly influenced by cancer-associated fibroblasts. To overcome the stromal contribution and explore cancer cell intrinsic features, we previously exploited gene expression profiles from CRC patient-derived xenografts (PDXs), devoid of human stromal signals, and identified five “colorectal cancer cell intrinsic subtypes” (CRIS), each endowed with unique molecular characteristics, drug response, and clinical outcome [11].

Despite their great potential for clinical applications, transcriptional classifiers tend to summarize biological traits into separate, non-overlapping subtypes. However, as already shown [12], subtype signatures may represent features with continuous degree of activity, not necessarily mutually exclusive, reflecting the complex phenotypes observed in human tumors. This is the case of glioblastoma in which multiple transcriptional subtypes were indeed observed to be activated in individual tumors [13, 14]. Likewise, in CRC, transcriptional heterogeneity for CMS was ascribed to both epithelial cancer cells and mesenchymal cell infiltrates [15, 16]. Moreover, subtype assignment typically relies on sets of samples [12, 17], which may lead to incoherent classification depending on the composition of the dataset. These observations, which are in line with histopathological evaluation, require a careful assessment to improve stratification and capture the biological nuances of human tumors.

To tackle these issues, we employed a large collection of human tumors and experimental models to explore the heterogeneity of CRC phenotypes, by fuzzy assignments and single-sample class prediction in bulk and single-cell profiling, unraveling the basis of multiple continuous phenotypes of CRC. These are the foundations for future clinical translation into transcriptome-based nomograms.

## Methods

### TCGA data

We used raw counts of RNA-seq expression data from the Colon adenocarcinoma (COAD) and Rectum adenocarcinoma (READ) projects of The Cancer Genome Atlas [18]. We downloaded this dataset from the GMQL repository [19] through its Web interface using the GMQL [20] query:

SELECT (gdc__project__project_id =  = "TCGA-COAD" OR.gdc__project__project_id =  = "TCGA-READ")

The dataset includes 698 primary tumor samples aligned to the GRCh38 human assembly, each with 58,387 profiled genes. Furthermore, we collected molecular and survival annotations for the TCGA dataset [21–23].

### Patient-derived xenografts collection

All the samples were obtained from patients treated by liver metastasectomy or primary tumor excision. Samples were procured and the study was conducted under the approval of the review boards of the institutions (protocol “Profiling”, code 225/2015 on 1 October 2015). Clinical and pathologic data were entered and maintained in our prospective database. All patients provided informed consent.

Tumor material not required for histopathologic analysis was collected and placed in medium 199 supplemented with 200 U/mL penicillin, 200 μg/mL streptomycin, and 100 μg/mL levofloxacin. Each sample was cut into 25- to 30-mm^3^ pieces in antibiotic-containing medium; 2 other pieces were coated in Matrigel (BD Biosciences) and implanted subcutaneously in 4- to 6-week-old female NOD (nonobese diabetic)/SCID (severe combined immunodeficient) mice, as previously described [24, 25]. At passage 2, multiple samples were subjected to gene expression profiling. In vivo experiments and related biobanking data were stored in the Laboratory Assistant Suite, a web-based, in-house developed data management system for automated data tracking [26]. All animal procedures were approved by the Ethical Commission of the Institute for Cancer Research and Treatment and by the Italian Ministry of Health (authorization 806/2016-PR), in accordance with Italian legislation on animal experimentation.

### Patient-derived xenograft RNA-seq profiles

We generated RNA-seq profiles from 646 liver metastatic and primary CRC PDXs [24] (each with 56,609 genes). To obtain bulk RNA-seq data, RNA was extracted using miRNeasy Mini Kit (Qiagen), according to the manufacturer’s protocol. The quantification and quality analysis of RNA was performed on a Bioanalyzer 2100 (Agilent), using RNA 6000 Nano Kit (Agilent). Total RNA was processed for RNA-seq analysis with the TruSeq RNA Library Prep Kit v2 (Illumina) following the manufacturer’s instructions and sequenced on a NextSeq 500 system (Illumina). Each generated FASTQ file was aligned using STAR 2.5.1 [27] and mapped to the human GRCh38 and Mus musculus GRCm38 genome reference combined. outFilterMultimapScoreRange was set to 3 to remove reads with ambiguous alignment to GRCm38. The GENCODE release 27 was used as transcriptome reference annotation, and gene expression quantification was performed with featureCounts [28, 29]. Data are available in Additional file 1: Table S1 and EGAS00001006492. Beside the RNA-seq data, we collected several metadata annotations such as KRAS, NRAS, BRAF, and PIK3CA gene mutations and the sensitivity to the Cetuximab drug treatment. Specifically, the latter one includes information on cancer volume variation after Cetuximab treatment and the sensitivity class, namely resistant (volume increase above + 35%), stable (volume variation between − 50 and + 35%) and sensitive (volume decrease greater than − 50%).

### TCGA and PDX RNA-seq data preprocessing

For the TCGA dataset preprocessing, we first extracted data from fresh samples only, to ensure uniformity with PDX samples that are not formalin-fixed paraffin-embedded. Additionally, we removed all samples for which the top 5 genes (i.e., the ones with the highest amounts of raw counts) account for at least the 20% of the sample total raw counts: indeed, these samples cannot be considered well-sequenced at the used sequencing depth. Moreover, for each patient, we kept a unique sample only, if needed choosing the one with the highest number of raw counts. Preprocessing of PDX batches included removing technical replicas, keeping only the samples with the highest number of raw counts. At the end of the preprocessing pipeline, we obtained 620 primary tumoral samples in the TCGA dataset and 606 samples in the PDX dataset, with a common set of 15,084 genes.

For both TCGA and PDX datasets, we computed the CPM (counts per million) expression values [27] from the raw counts using the CPM function of the edgeR package [30]; next, we focused only on the genes of the CRIS signature [11], which characterize each CRIS class and are here used as features of all the proposed classifiers.

### CRC single-cell data and preprocessing

Public single-cell RNA-seq data were downloaded from the NCBI Gene Expression Omnibus (GEO) database under the accession code GSE132465 [31]. These data regard 63,689 cells obtained from 23 patients with primary colorectal cancer. To distinguish and select the epithelial tumor cells, we used the SingleR [32] tool, which uses as reference transcriptome features of several pure cell types. From the epithelial cancer cells, potential doublets were removed using the DoubletFinder tool [33]. Then, we filtered out low-quality cells with less than 1000 genes supported by at least 4 reads. Furthermore, since the SMC05 and SMC15 patients were represented by less than 20 cells, we excluded them from further analyses. In total, 4291 cells passed all the described criteria.

### Patient-derived organoid bulk/scRNA-seq data and preprocessing

We generated scRNA-seq and bulk profiles for an in-house collection of 5 CRC patient-derived organoids (EGAS00001006214, PDO). The PDO single-cell profiles were generated by 10xGenomics, obtaining 15,766 single-cell profiles across all 5 organoid samples. To remove the potential doublets, we used the DoubletFinder tool [33]. Then, we filtered out low-quality cells with less than 1000 genes supported by at least 4 reads. In total, 4616 cells passed all the described criteria. Instead, the PDO bulk profiles were performed by poly(A) RNA capture (Illumina) with more than 12 million reads per sample and 16,183 transcripts detected with at least 4 reads. After preprocessing, the scRNA-seq and bulk PDO profiles were normalized using the CPM approach [33].

### Pseudo-bulk from scRNA-seq data

The pseudo-bulks of GSE132465 [31] and PDO scRNA-seq data have been generated by summing the gene counts detected in all the cells belonging to the same patient. For the calculation, we used the cells passing all the criteria previously described. The pseudo-bulk counts of tumors and organoids were normalized using the CPM method [34].

### CRIS classification

We employed, as a reference, the original CRIS classifier based on the Nearest Template Prediction algorithm (NTP) [35], which returns the closest class template for each sample. This CRIS classifier uses a dataset-wide computation of the expression *Z*-score value for each gene in each sample: these *Z*-score normalized profiles are then compared with the five CRIS class templates (i.e., centroids) to assign the sample to the class whose centroid is at the minimum significant distance from the sample normalized profile [11]. The threshold chosen for significant sample classification was the Benjamini–Hochberg false discovery rate (BH.FDR) < 0.2, as previously reported [2, 11, 35]. Accordingly, NTP-based classification was applied to the above described bulk, single-cell, and pseudo-bulk RNA-seq profiles. In particular, we developed a new NTP-based multi-label implementation of the CRIS classifier, “multiCRIS,” able to assign each sample to one or more CRIS classes based on the distance from each CRIS centroid and on its significance (https://github.com/cisella/multiCRIS, Supplementary material Sect. 2.3).

For single-cell data, all genes with at least one read per gene in a sample were included, for a total of 95% of the CRIS gene signatures. For the implementation of single-sample classifiers on the CRIS classification task, we used only confident NTP single-label assignments as state-of-the-art references for the training and testing phases of each alternative method; specifically, this meant using 562 confidently classified TCGA samples and 550 confidently classified PDX samples.

### Single-label classifiers

We implemented and compared the performances of several state-of-the-art single-label classifiers to assign the most prominent CRIS class (primary class) to each sample individually: Random Forest (RF) [36], Extreme Gradient Boosting (XGBoost) [37], Neural Networks (NN; with a single hidden layer) [38], and Support Vector Machines with either linear (LSVM), polynomial (PSVM), or Gaussian radial basis function (GRBF-SVM) kernel [39].

Using NTP single-label assignments as target references and CRIS gene signature as feature space, we trained all the assessed classifiers with a tenfold stratified cross-validation on 70% of the TCGA confident samples (BH.FDR < 0.2); the remaining 30% was instead kept aside for a first testing. We included 393 TCGA samples in the training set and 169 TCGA samples in the testing set, while all 550 PDX samples were used entirely as an independent testing set to further evaluate the performances of all the classifiers on completely independent data of a different type (liver metastasis PDXs instead of primary human tumors). During the training phase, we also performed hyperparameter tuning to optimize the performances of the classifiers by finding the best hyperparameter values, then used to train each model one last time on the entire training set.

All the details about the mentioned models, data splitting, hyperparameter tuning, and training Supplementary and testing phases are described in Supplementary material (Sects. 3.1–3.2), together with the metrics used both to cross-validate and test each model of interest (Sect. 3.4). Additionally, we compared the obtained results with the performances of the Top Scoring Pairs algorithm [11], the only already existing attempt of a single-sample CRIS classifier.

### Multi-label single-sample classifiers

To move towards multi-label classification, we made use of algorithm adaptation strategies [37]; this kind of approach modifies existing single-label models to cope directly with the multi-label setting, as done also for the NTP classifier to obtain the multiCRIS classifier. While the primary class is directly inherited from the corresponding single-label classifiers, we worked on the scores of the single-label models to adapt them to cope with multi-label assignments. This approach resulted to be really promising in dealing with the CRIS classification in a multi-label scenario; in Supplementary material Sect. 3.3, we reported our algorithm adaptation procedure in full detail.

### MultiCRIS references for testing single-sample multi-label classifiers

To assess the performances of our multi-label single-sample classifiers, we used multi-label calls coming from our multiCRIS approach. In multiCRIS, a sample can be assigned to one or more “secondary” classes for which the membership is lower than for the primary class but still significant (BH.FDR < 0.2). To define reliable secondary classes as reference targets, the NTP multiCRIS calls of each sample were further compared to class-specific thresholds. These thresholds were computed for each class as the 5th percentile of the primary class NTP scores on the entire TCGA and PDX sets separately (all details are reported in Supplementary material Sect. 2.3). This procedure confirms the primary assignment of the NTP single-label, and possibly assigns secondary classes that must be confident calls but also exceed a minimum accepted membership score. The so-obtained multiCRIS calls were used as reference targets for testing single-sample, multi-label models.

### Clinical and biological evaluation

Clinical and biological validations have been performed on the results provided by the assessed classifiers using survival analysis with Kaplan–Meier curves [40] and Fisher statistical tests [41], as detailed in Supplementary material Sect. 3.5.

We used Kaplan–Meier curves computed for the first 36 months, due to the high number of censored patients (i.e., for whom the follow-up has terminated and thus no outcome information is available after a given date) between 36 and 60 months in the considered datasets. Because of this, all samples with disease-free survival greater than 36 months have been considered as disease-free at the 36-month endpoint. To assess the statistical significance of the difference between two compared survival distributions, we used the log-rank test, a non-parametric hypothesis test that can be used also when some observations are censored.

For Fisher tests, we used a *p*-value threshold of 0.05 to discriminate statistically significant annotations from not significant ones. Also, to enrich our evaluation of each clinical correlate, we computed odds ratio and effect sizes, which respectively make comparisons with the overall scenario or with the complementary cases, as detailed in Supplementary material Sect. 3.5.

## Results

### Multi-label CRIS stratification of colorectal cancer

To improve stratification and capture the biological traits of CRC according to the CRIS classification, we reasoned that its Nearest Template Prediction (NTP) algorithm can be employed not only to assign the single most prominent class, but also to evaluate the assignment of each sample to all CRIS classes, as well as the false discovery rate of each assignment [11]. Thus, we implemented a new NTP-based multi-label version of the CRIS classifier, “multiCRIS,” able to assign each sample to one or more CRIS classes based on the distance from each CRIS centroid and on its significance (Supplementary material Sect. 2.3).

**Fig. 1:**
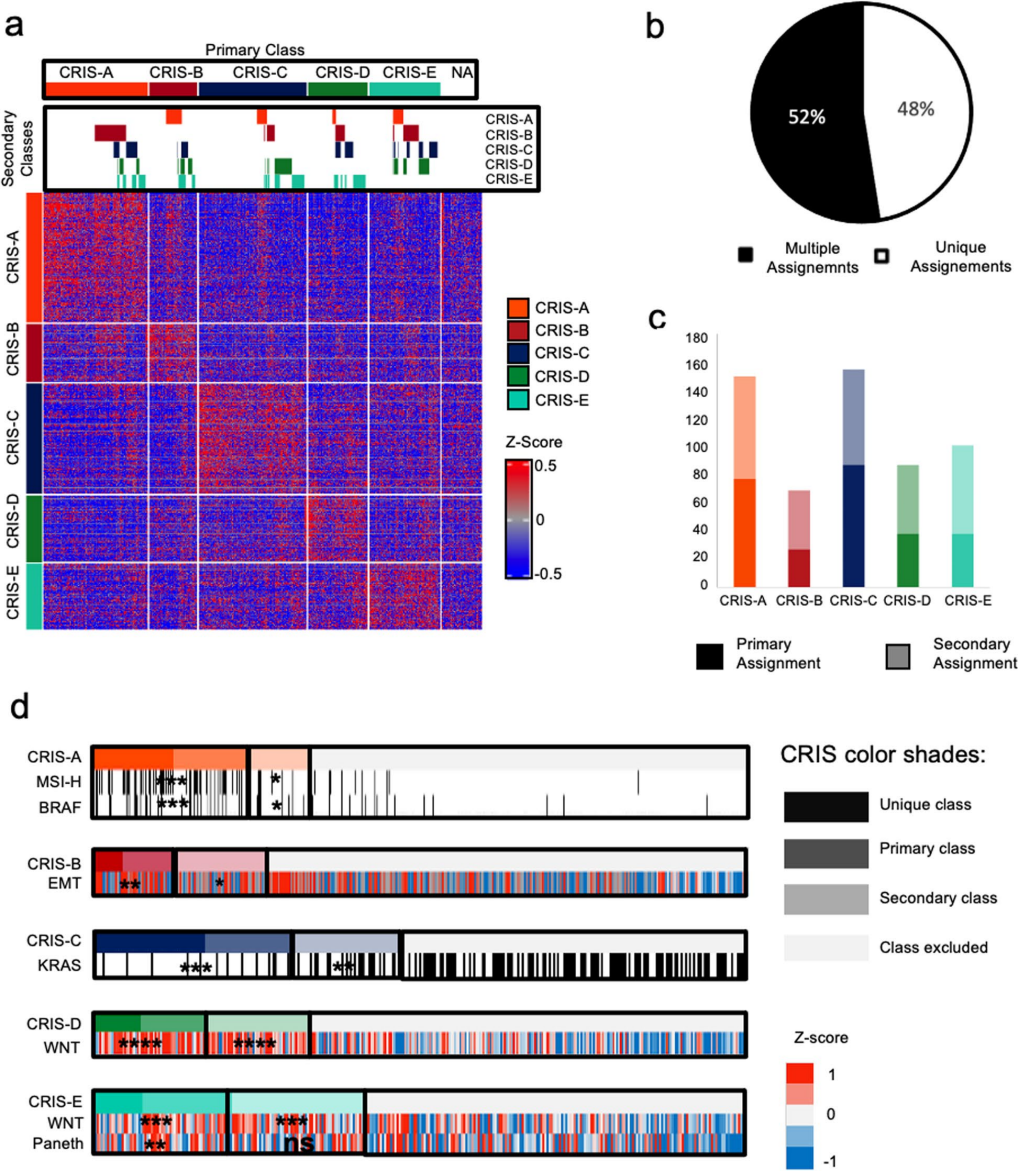
Multi-label CRIS classification of 596 CRC samples from TCGA. **a** Heatmap representing CRIS classes genes (rows) as *Z*-scores and classified samples (columns). Above the heatmap, a panel reports secondary classifications. **b** Pie chart representing the proportion of samples with unique (white) or multiple (black) class assignments by multiCRIS in the TCGA CRC dataset. **c** Proportions of primary and secondary assignments for each CRIS class in the TCGA dataset. **d** Heatmap representing the enrichment of key molecular features in primary and secondary CRIS classes; black bars indicate positivity to a molecular feature (i.e., MSI status, and BRAF/KRAS mutations); *Z*-scores are represented for continuous variables such as EMT, Paneth and WNT scores. ***p-value < 0.0001, **p-value < 0.001, *p-value < 0.05, ns = not significant

MultiCRIS was first applied to the 620-sample RNA-seq dataset from The Cancer Genome Atlas (TCGA) [18], to confidently assign 91% of the samples to at least one class (Fig. 1a, Additional file 2: Table S2, BH. FDR < 0.2) [11]. Interestingly, 52% of the samples could also be confidently assigned to additional CRIS subtypes (Fig. 1b). To verify if different FDR thresholds could impact on multiple class assignment, we performed multiCRIS analysis with more stringent thresholds and found that the fraction of samples belonging to multiple classes was only marginally reduced (Additional file 3: Table S3).

Therefore, each tumor may be assigned either to a single class (48% of the assigned samples), to whom it displays the only significantly low distance, or to multiple classes (Fig. 1b), of which the class with the lowest distance is the primary.

Notably, for all CRIS subtypes, the number of secondary assignments was grossly equivalent to primary assignments (Fig. 1c, Additional file 4: Table S4). As expected, non-primary assignments displayed significantly higher distance to the CRIS centroids (Additional file 5: Table S5). However, multiple assignments occur preferentially within two specific subfamilies: CRIS-A/CRIS-B and CRIS-C/CRIS-D/CRIS-E as previously described [11] (Additional file 6: Table S6). Finally, to assess whether these multiple assignments captured tumors with multiple CRIS biological traits, we explored the main characteristics associated with each CRIS class.

Interestingly, samples assigned to secondary classes mostly retain key molecular features of the classes as shown in Fig. 1d, including MSI status for CRIS-A, depletion of KRAS mutations in CRIS-C, together with WNT pathway activity in CRIS-D/CRIS-E and Epithelial Mesenchymal Transition (EMT) in CRIS-B samples (Additional file 7: Table S7). Notably, we observed that samples with multiple assignments tend to have higher distances from CRIS centroids, which could reflect either a composition of cells concomitantly harboring different phenotypes or a mixture of cells with different phenotypes.

### Single-cell heterogeneity in multiple CRIS assignments

The observed multiple class assignment of a consistent fraction of CRCs could be explained in two ways: tumors are composed of cancer cells with ambiguous phenotype, or mixed populations of cells of different subtypes are present. To explore the heterogeneity underpinning multiCRIS assignments, we performed a set of paired single-cell RNA sequencing (scRNA-seq) and bulk profiles in an in-house collection of 5 CRC organoids derived from PDXs. These data allowed direct comparisons of single-cell and bulk transcriptional profiles. As a third option, pseudo-bulk profiles were obtained by aggregating all single-cell profiles derived from one sample. Notably, while the profiles from individual cells captured on average 1116 transcripts with at least 5 supporting reads (Additional file 8: Figure S1), the pseudo-bulk profiles traced more than 17,095 transcripts on average (Additional file 9: Table S8a). As expected, the profiles from matched bulk/pseudo-bulk samples displayed strong correlations, which could not be reached by unmatched comparisons (average correlation *R* = 0.684, R-test *p*-value < 2.20^−16^; Additional file 8: Figure S2). These results indicate that (i) single-cell profiles display high heterogeneity and (ii) aggregated single-cell profiles recapitulate the transcriptome obtained in bulk profiles. Thus, the 3D in vitro organoid culture system captures a complex spectrum of transcriptionally heterogeneous cells.

**Fig. 2:**
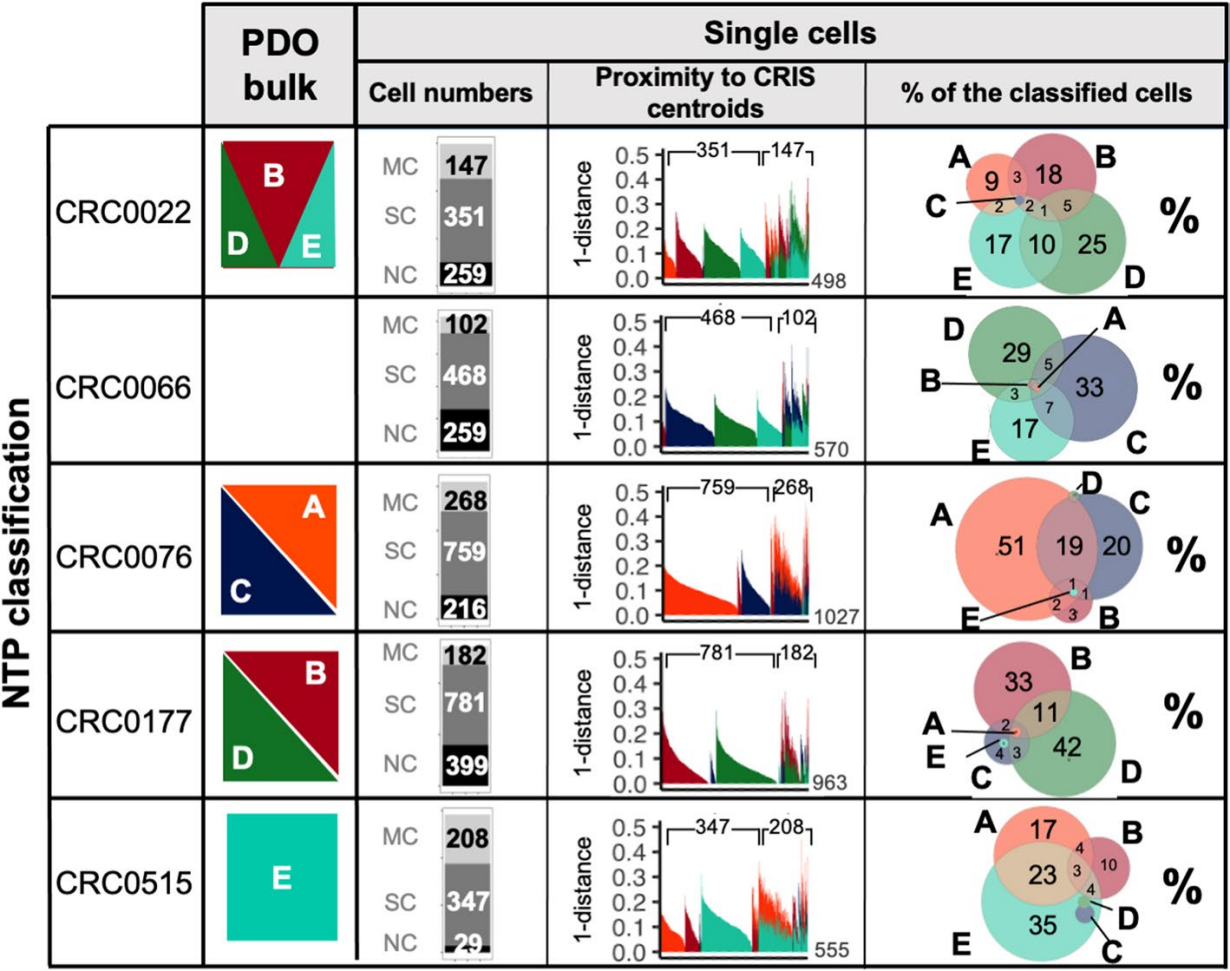
MultiCRIS classification of human organoids. For each organoid line from left to right they are reported: MultiCRIS classification of bulk RNA-seq profile; number of cells with multiple assignments (MC), single assignment (SC), and not classified (NC); proximity of each classified cell to one or more centroids. Venn diagrams revealing the intersection between CRIS classes at the single-cell level

In line with previous results, the CRIS classification of organoids’ bulk RNA-seq profiles revealed multiple class assignments for three organoids, a single class assignment for one organoid, and one non-classified organoid (Additional file 10: Table S9). To explore whether multiple class assignments are due to the presence of cells with a hybrid phenotype rather than a mixture of cells with different phenotypes, we performed CRIS assignments on single-cell profiles (see “Methods” section and Additional file 11: Table S10). Interestingly, 78% of single cells were successfully assigned to at least one CRIS class; of these, 75% were assigned to a single class and 25% to multiple CRIS classes (Additional file 11: Table S10). Notably, we found both coexisting mixtures of cells, each with a single CRIS assignment, and cells with hybrid multiCRIS classes. Individual cells from a given organoid were mainly assigned to the CRIS class/classes defined by the bulk profile of that organoid (Fig. 2).

These results highlight that, at single-cell resolution, most cells are assigned to individual CRIS subtypes and that their mixture is responsible for multi-class assignment of the bulk transcriptome; however, it is also possible that a small but sizable portion of cells with a hybrid phenotype may contribute to the assignment of multiple CRIS classes to a given bulk sample. Indeed, we detected the coexistence of both cells with different CRIS identity and cells with a hybrid phenotype in all the organoids that received multiple CRIS bulk assignments (Fig. 2).

**Fig. 3:**
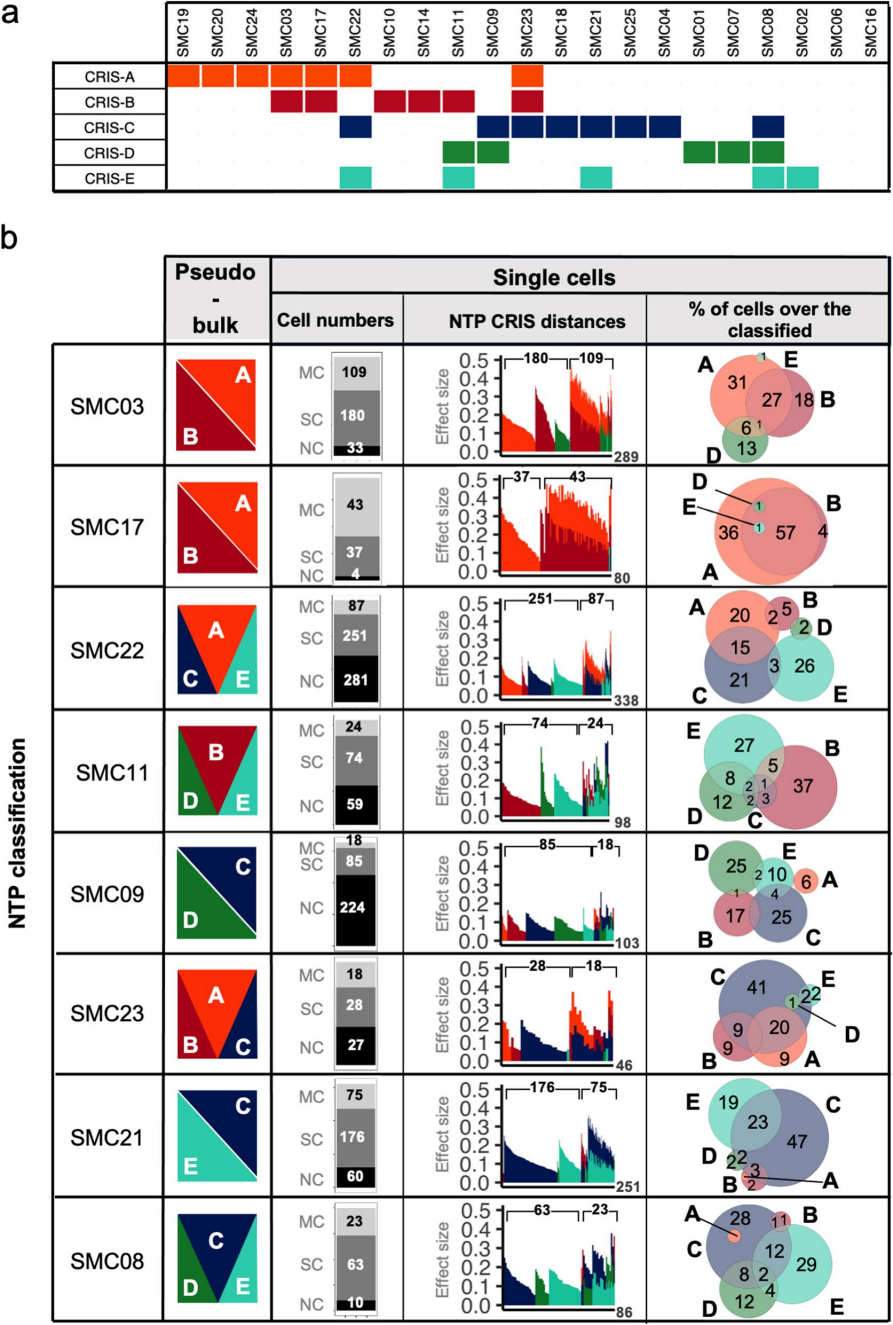
MultiCRIS classification of human CRCs. **a** multiCRIS classification of human pseudo-bulk tumors derived from single-cell profiles. **b** For each CRC case, from left to right they are reported: MultiCRIS classification of the pseudo-bulk profile; number of cells with multiple assignments (MC), single assignment (SC) and not classified (NC); proximity of each classified cell to one or more centroids. Venn diagrams revealing the intersection between CRIS classes at the single-cell level

To extend our observation to human tumors, we took advantage of public scRNA-seq data from a cohort of patients (GSE132465) [31], focusing on epithelial cells to compare multi-label CRIS assignments of pseudo-bulk and single-cell profiles: such analysis confirmed the occurrence of patients harboring multiple CRIS assignments (Additional file 12: Table S11). In these samples, we confirmed that most individual cells are assigned to a specific CRIS class (64% of classified cells, of which 75% assigned to a single CRIS class and 25% to multiCRIS groups; Fig. 3a; Additional file 8: Figure S3; Additional file 13: Table S12). However, similarly to organoids, each sample was composed by different cell populations classified into various CRIS subtypes, leading to a complex phenotype that was captured by multiple CRIS assignments of pseudo-bulk profiles (Fig. 3b). Accordingly, samples assigned to a single CRIS class tended to have a higher proportion of cells assigned to that class (Additional file 8: Figure S3). In specific samples, the high percentage of individual cells with multi-label assignments may reflect a portion of tissue undergoing a functional switch or a stable intermediate differentiation stage. For example, this occurred in patient SMC17 (Fig. 3b), in which 57% of classified cells displayed multi-label phenotypes. Similarly, SMCO3 and SMC21 patients showed 34 and 28% of cells, respectively, with hybrid phenotype (Fig. 3b) in line with their multi-label status traced in bulk profile.

Altogether, these results show that the heterogeneity of the CRIS transcriptional profile is rooted at the single-cell level and that the phenotypes of individual cells sum up to define the CRIS classification of the bulk tumor. Therefore, the evidence of multiCRIS tumors can be mainly explained either by a mosaic composition of different cell populations with specific functional characteristics, or by a small portion of hybrid cells with mixed phenotype.

### Single-sample approaches for CRIS classification

**Fig. 4:**
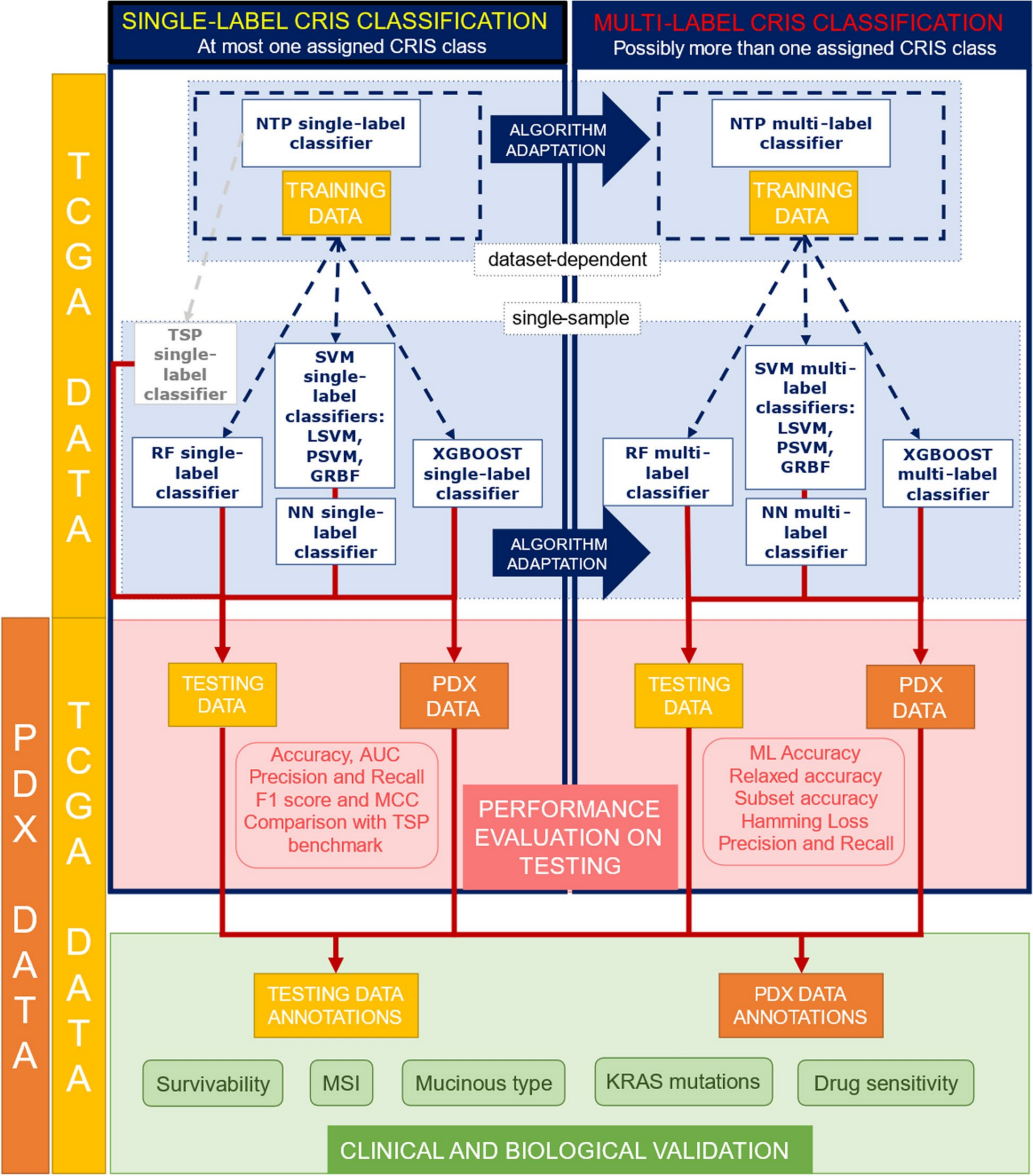
Workflow for machine learning-based construction of a single-sample CRIS classifier. Overview of the main steps (training, testing, and clinical/biological validation) on TCGA and PDX CRC samples, for single-label and multi-label CRIS classifier construction

MultiCRIS paves the way for complex biological and clinical readouts; however, it is influenced by its NTP implementation, which relies on centroid distance and gene-level *Z*-score calculated on batches of samples, without allowing single-sample classification [35]. To overcome this, we moved towards single-sample algorithms, able to classify each sample independently: these algorithms can deal both with single-label assignment to the primary class only (SC), or with multi-label assignments (MC), to capture inner heterogeneity. Our workflow is outlined in Fig. 4; it includes an initial training phase for all the algorithms (in blue), their performance evaluation on testing data (in pink) and a final clinical and biological validation of the most promising single-sample approach (in green). We first implemented single-sample single-label algorithms able to recognize the most prominent (primary) class of each sample, as the original version of the NTP classifier does for dataset-dependent classifications [11]. The considered approaches include Random Forest (RF) [36], Support Vector Machines with Linear (LSVM), Polynomial (PSVM), and Gaussian Radial Basis Function (GRBF-SVM) kernels [39], Neural Networks (NN) [38], and Extreme Gradient Boosting Trees (XGBoost) [37]. These single-sample algorithms were subsequently adapted to the multi-label context by extracting all CRIS class memberships of each sample; this allowed the validation of their results against those of the multi-label NTP (multiCRIS).

To evaluate each algorithm and identify the one most suitable to predict CRIS class memberships in a clinically applicable single-sample classifier, we took advantage of the collection of primary CRC samples from the TCGA project (*n* = 562) and of the cohort of patient-derived xenograft (PDX, *n* = 550). TCGA data were divided in training and testing sets, keeping the same CRIS class proportions of the entire dataset. Each classifier was trained considering only the expression values of the CRIS genes as feature space and using the NTP primary class as target reference [11], regardless of its single- or multi-label usage. A 30% of the TCGA samples and the full PDX dataset were used as two independent testing sets to evaluate the results of single-label and multi-label classifiers.

**Fig. 5:**
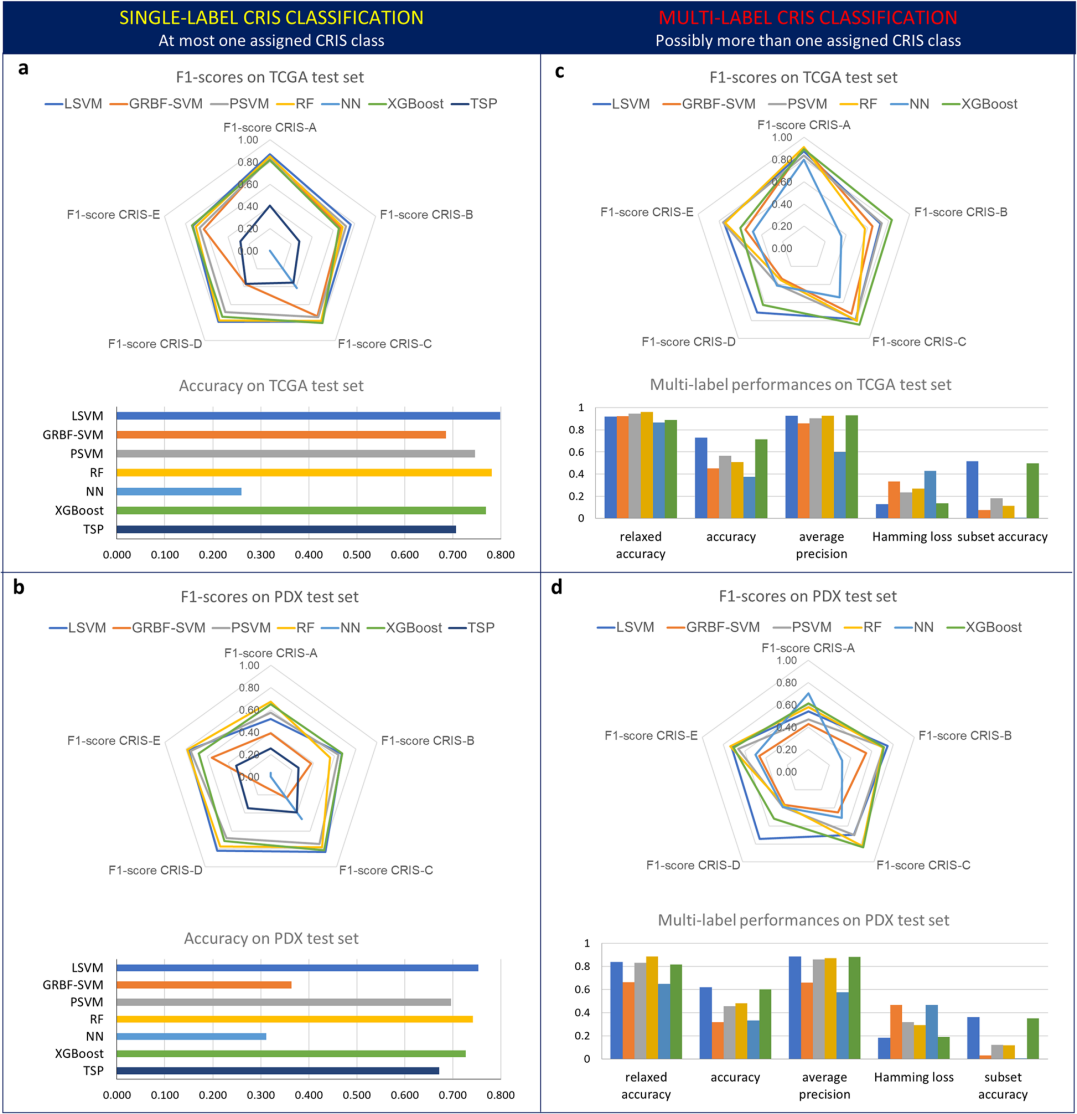
Performance evaluation of machine learning-based CRIS classifiers. For each CRIS class, F1-scores, and additional performance metrics (see text) for different machine learning classifiers are reported for single-label (**a**, **b**) and multi-label (**c**, **d**) configurations, on the TCGA test set (**a**, **c**) and PDX validation set (**b**, **d**)

In single-label evaluation, focused on primary class assignments, we employed global accuracy, precision, and recall of each class, derived metrics (F1-score and Matthews correlation coefficient (MCC)), and threshold-based measures (areas under the receiver operating characteristic and the precision-recall curves), as to evaluate the performances of the considered algorithms and compare them with the ones of the original NTP approach [35] (Additional file 14: Table S13). LSVM reached approximately 80% of accuracy on TCGA testing and 75% on PDX, showing good performances when considering both precision and recall of all the classes (Fig. 5). Although XGBoost and RF achieved interesting performances, their class-specific behaviors appear less stable and slightly worse overall than those of the LSVM (Fig. 5).

Furthermore, in the single-label scenario, the machine learning methods were compared with the TSP approach, a first attempt of a single-sample CRIS classifier, that had originally shown quite limited concordance with NTP classification [11]. Its sub-optimal results are confirmed in the current study: all our classifiers achieve higher accuracies compared to TSP, which reached at most 70.7% (Additional file 14: Table S13). Particularly for LSVM, even the class with worse F1-score on the TCGA test set (CRIS-E, with 73%) significantly overcomes the TSP result (CRIS-E with 28%).

Also, on the PDX set, LSVM reaches the most convincing results. The graphs in Additional file 8: Figure S4a and S4b show similar distributions of the CRIS classes within the two TCGA testing and PDX datasets. They indicate that, in TCGA, CRIS-A is the most frequent class, being slightly more represented than CRIS-C; CRIS-E follows, while CRIS-B and CRIS-D are lower in size but comparable. Almost the same trend can be noticed in PDX, with the only exception of the CRIS-A class that is underrepresented in PDXs as a consequence of the scarcity of MSI cases (for which CRIS-A is enriched) among samples coming from metastatic CRC [23].

Thus, based on performance evaluations, we identified LSVM as the best single-label classifier in predicting the primary CRIS class of each single CRC sample. However, all the three trained algorithms are capable of computing memberships to all the 5 CRIS classes by means of algorithm adaptation techniques [42], which paves the way towards the multi-label context.

### Multi-label CRIS classification through single-sample approaches

Following an algorithm adaptation strategy, we developed multi-label adapted (mla) single-sample CRIS classifiers. Specifically, each mla algorithm inherits the primary class assignment from its single-label version but can associate any heterogeneous sample with one or more additional secondary classes.

To evaluate mla classifiers, we used both metrics analogous to the single-label ones, but adapted for the multi-label context (relaxed accuracy, precision, recall) and specific multi-label measures (average precision, Hamming loss, subset, and multi-label accuracy). All these metrics compare the results of mla algorithms with target assignments obtained from the MultiCRIS approach, introduced in this work.

Among mla algorithms, LSVM still reached the best overall performance when considering class precisions and recalls, revealing to be the most robust approach also in the multi-label context (Fig. 5, Additional files 15, 16, 17: Tables S14, S15, S16). Furthermore, LSVM assigned the primary multiCRIS class (i.e., the most prominent class according to the NTP algorithm) in 91.7% of the cases (relaxed accuracy) and reached an average precision of 92.6% in predicting the multi-label characterization of the TCGA testing samples. When considering the Hamming loss, which represents the average fraction of misclassified labels, LSVM was the approach having the lowest fractions, both in TCGA testing and PDX sets. Eventually, LSVM subset accuracy (the strictly identical attribution of all the expected labels) was quite relevant, even more considering that each algorithm is trained by providing the primary class only as a reference target.

Thus, LSVM clearly emerged as the best approach to perform single-sample classification, either on a single-label or multi-label perspective. The distributions of the CRIS classes predicted by the single-label (a, b) and multi-label adapted (c, d) LSVM on the TCGA testing and PDX sets, respectively, are reported in Additional file 8: Figure S4. Multi-label distributions are coherent with expectations: in both the datasets, CRIS-C results to be the most prevalent class, while CRIS-B doubles its assigned samples. Conversely, CRIS-D and CRIS-E collect more non-primary assignments on PDX than on TCGA; eventually, CRIS-A is the second most frequent in TCGA while stays still underrepresented in PDX, as discussed for the single-label case.

### Clinical and biological evaluation of single-label and multi-label LSVM classifier

Both the single-label and multi-label LSVM classification results were evaluated for molecular and clinical correlates.

**Fig. 6:**
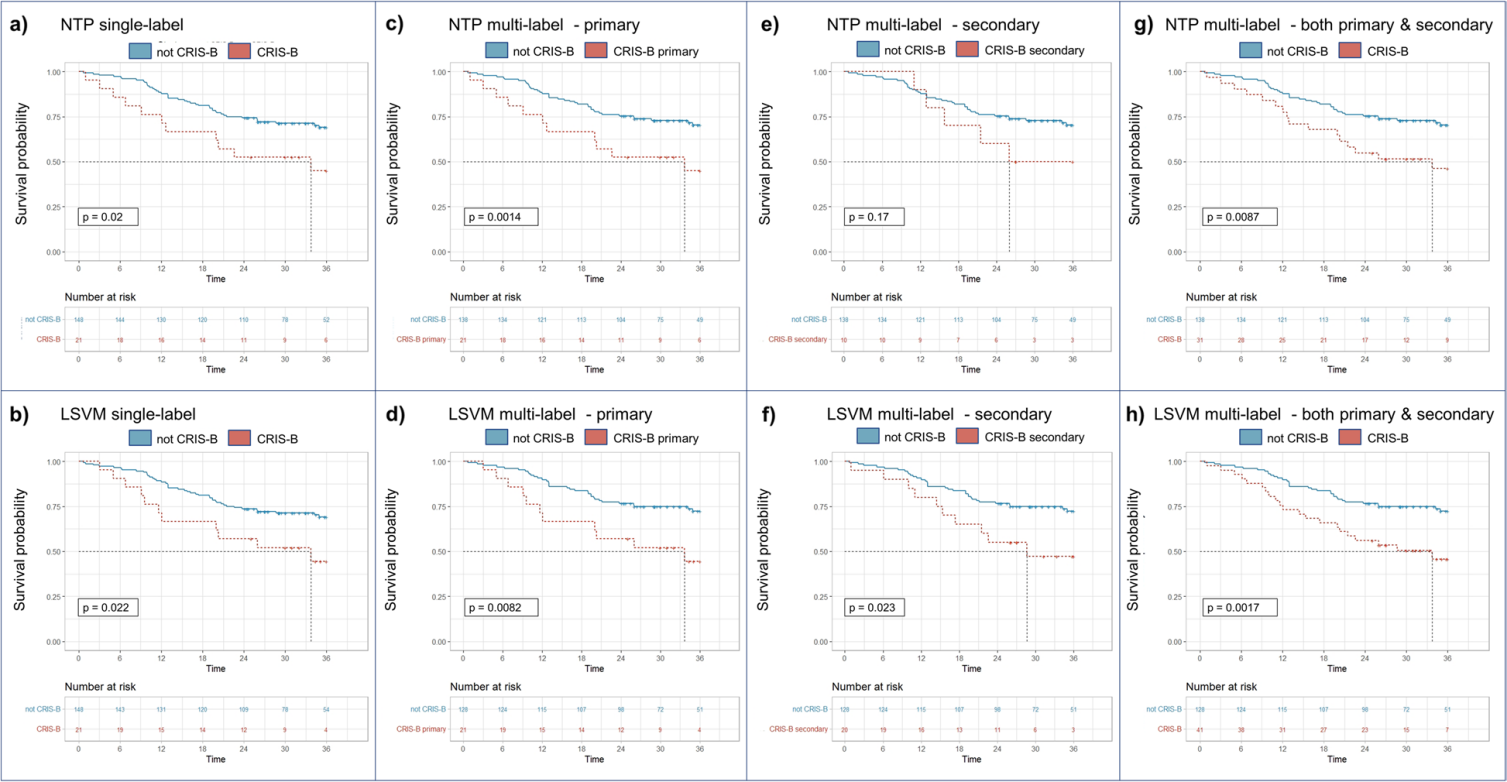
Prognostic significance of single- and multi-label CRIS-B assignment. Kaplan–Meier survival curves over 36 months focused on CRIS-B classification by NTP-based and machine learning-based methods in the TCGA CRC dataset. The prognostic value of CRIS-B assignment by NTP (top) and LSVM (bottom) is displayed for **a, b** single-label CRIS-B vs non-CRIS-B; **c, d** CRIS-B primary vs. non-CRIS-B, excluding cases with secondary CRIS-B assignment; **e, f** CRIS-B secondary only vs non-CRIS-B; **g, h** CRIS-B primary plus secondary vs non-CRIS-B

We first evaluated the prognostic value of LSVM-based models, on the TCGA dataset considering different scenarios: only the samples assigned to the class as primary class, only the samples assigned to the class as secondary class, or all the tumors classified to the CRIS class regardless of whether it is a primary or secondary assignment. In all such cases, any comparison using the Fisher test [43] is always with regard to all the samples that are not assigned at all to the CRIS class under examination. For both the NTP and LSVM-based single-label classifiers, Kaplan–Meier (KM) [40] survival analysis confirmed that the CRIS-B class is significantly associated with poor prognosis (Fig. 6a,b). Interestingly, with multi-label assignments, excluding samples with secondary CRIS-B assignment from the non-CRIS-B group highlighted an even higher association with poor prognosis (Fig. 6c,d). Accordingly, when samples with primary CRIS-B assignment were excluded from the analysis, samples with secondary assignment to CRIS-B displayed worse prognosis (Fig. 6e,f). When primary and secondary CRIS-B cases were joined, prognostic significance reached the maximum values (Fig. 6g,h). Notably, prognostic significance was higher for LSVM-based classification in all cases.

Response to anti-EGFR treatment was evaluated in the PDX cohort. We confirmed associations of CRIS-C and sensitivity to Cetuximab, for which single-label LSVM (odds ratio (O.R.) = 3.281, confidence interval (CI) = 1.66–6.73) and multi-label LSVM, including also secondary assignments in the CRIS-C cohort, displayed similar performances (O.R = 3.36, CI = 1.24–10.64) (Additional file 8: Figure S5).

Other molecular and clinical characteristics of the CRIS subtypes, already extensively analyzed and disclosed by Isella et al. in [11], were significantly captured with LSVM models, either considering the primary class only, or the complete multi-label characterization, as shown in Additional file 8: Figure S5. This is particularly evident for the enrichment of MSI-high cases in CRIS-A and for the depletion of KRAS mutations in CRIS-C samples. Additionally, we performed a feature importance analysis based on the coefficients of the LSVM models to prioritize and extract the most important genes for each class. This selection highlighted genes with interesting roles and functions for each class, summarized in Additional file 18: Table S17. In particular, for CRIS-A: FCGBP, an immunoglobulin-binding mucin involved in humoral immune responses in CRC [44], and ANKRD37, induced by hypoxia and likely involved in autophagy [45]; for CRIS-B: Kallikreins 10 and 6, and IGF2BP3, all known to promote cancer metastasis [46–49], consistently with the poor prognosis of the CRIS-B subtype; for CRIS-C: two metabolic enzymes, CES2 and QPRT, both involved in promoting resistance to antineoplastic agents [50–52], highlighting CRIS-C-specific possible therapeutic strategies; for CRIS-D: three genes involved in the WNT pathway, enhanced in CRIS-D, either as a modulator (NOTUM) [53], a target (FGF20) [54] and a non-canonical mediator (PTK7) [55]; and for CRIS-E: PTPRO, a tyrosine phosphatase receptor induced by the WNT pathway [56], typically active in CRIS-E subtype.

All these evidences confirmed the reliability of the predictions obtained with our LSVM single-sample models, especially the mla LSVM, henceforth referred to as ML^2^CRIS (*M*ulti-*L*abel *M*achine *L*earning CRIS, which is able to highlight biologically meaningful inner heterogeneity of the samples (if any), while assessing each patient individually in a clinical usage setting.

## Discussion

The global transcriptome profile of bulk tumors provides and aggregates portraits of several cell types composing the whole tumor ecosystem, including cancer cells, vessels, fibroblasts, and immune cells. These data provide invaluable information to discern the biology of different tumor types and improve patient stratification for clinical practice. However, such data stem from different tissues, and in current biomedical research, it is essential to dissect the contribution of each cell type to tailor the most suited therapeutic strategy.

In this perspective, the CRIS classifier was designed to subdivide CRC into five subtypes specifically based on the intrinsic epithelial cancer cell transcriptome [9, 11]. This cancer cell-oriented taxonomy not only assists in stratifying patients by outcome, it also captures different dysregulated biological traits, which can be targeted for novel therapeutic strategies, independently of the amount and composition of the stromal cell compartment [57]. However, the CRIS classifier required two key implementation aspects to be solved. First, NTP-based classification univocally assigns one subtype label to a tumor, ignoring the potential coexistence of traits pertaining to different subtypes. Indeed, the membership of a sample to a transcriptional class is a quantitative and probabilistic attribute, rather than a qualitative, univocal one [12, 17]. Furthermore, a tumor may display ambiguous transcriptional features, associated with more than one subtype and still informative for the prediction of specific biological and clinical features as previously show in glioblastoma [13, 14] and CRC for CMS classifier [15, 16]. Second, NTP relies on a large cohort of samples to properly standardize the data and assign a tumor to a CRIS class. To improve feasibility in clinical practice, a novel implementation of CRIS subtyping should allow profiling and classification of individual samples, while maintaining the capacity to explore the intratumoral heterogeneity of CRIS classes.

In this work, we show that multiple CRIS subtypes can coexist in the same tumor and that this is a quite common occurrence in CRC. To investigate this aspect, we implemented a multi-label CRIS NTP classifier to enable statistically significant assignment of each tumor sample to one or more classes. With multi-label classification, the majority of TCGA CRC cases received multiple assignments. The observation that key biological and molecular features were associated with CRIS classes, even when a secondary assignment was attributed to the sample, indicates that a fuzzy classification reliably reflects the complexity of the tumor biology. Multiple assignments were driven by two main causes: (i) concomitant presence of cell subpopulations with distinct CRIS phenotypes, or (ii) a homogeneous cell population carrying a hybrid CRIS phenotype. To discern the relative contribution of each of the abovementioned drivers of heterogeneity, we took advantage of matched single-cell and bulk RNA-seq profiles to compare the fuzzy assignment of the tumor bulk with single-cell multi-label CRIS assignments. The observed concordance of classification between organoid bulk and single-cell RNA-seq profiles confirmed the adequacy of multiCRIS also for single-cell classification. It should be noted that the robustness of multiCRIS derives from its use of lists of signature genes, without any quantitative parameters assigned to them. This approach has proven robust to cross-platform analyses, which typically involve dropout of signature genes [35]; furthermore, CRIS classifier genes were initially selected for having highly variable expression and therefore be highly expressed in at least a subset of samples [11]. This analysis revealed that, in most cases, coexistence of cells assigned to distinct CRIS subtypes is likely to explain the multiple assignments of the bulk profile. However, we also observed cells in which multiple CRIS traits coexist, and, in a minority of cases, cells with a hybrid CRIS phenotype were the dominant population. These observations are in line with a previous work describing intratumoral heterogeneity of the CMS subtype at the single-cell level: Lineage-dependent gene expression programs influence the immune landscape of colorectal cancer [31]. The new multiCRIS approach proposed in this work allows the exploration of fuzzy membership in CRIS transcriptional classes capturing intratumoral heterogeneity. Future characterization of these new mixed phenotypes will allow defining their stable or transitory nature, increasing the understanding of tumor evolution and cell differentiation.

To overcome the need of NTP for a large sample series, machine learning-based single-sample classification methods (SVMs, NN, RF, and XGBoost) have been evaluated here. These methods can reflect sample inner complexity while overcoming the dataset-dependence issue of the multiCRIS NTP approach. This is essential towards a clinical application of such transcriptional classification of patients’ samples, single or in small batches, to assist in therapy decision in clinical practice. Furthermore, this method can also be successfully applied to data from clinical trials, which typically target a specific subgroup of cases that are likely not balanced in terms of CRIS class representation. Among all the tested classifiers, the Linear Support Vector Machine resulted as the best algorithm, both in single-label and in its multi-label adapted configuration, named ML^2^CRIS. Indeed, the here designed multi-label adaptation procedure allows enriching the original single-label assignments of the model with the addition of secondary classes. ML^2^CRIS correctly assigns the CRIS classes based on the original single-label NTP algorithm in 91.7% of TCGA test cases, with an average precision of 92.6% in estimating the multi-label characterization of each sample. Besides these encouraging performances, primary and secondary CRIS assignments of ML^2^CRIS confirmed that the molecular traits characterizing each of the five subtypes (e.g., enrichment of KRAS mutations and MSI-high status for CRIS-A, depletion of KRAS mutations for CRIS-C, and poor prognosis of CRIS-B) were maintained also in secondary assignments. When the single-label LSVM-based classifier was applied, CRIS-C cases were significantly associated with Cetuximab sensitivity (OR = 3.281, *p*-value < 0.0003). In the multi-label scenario of ML^2^CRIS, when secondary CRIS-C cases were added to primary ones, the odds ratio increased (3.359), although with reduced significance (*p*-value = 0.0127). However, when secondary CRIS-C cases were removed from both groups, and CRIS-C primary cases were compared with non-CRIS-C (not even secondary), the odds ratio increased together with its significance (OR = 4.164, *p*-value < 0.004).

Therefore, ML^2^CRIS shows promising capabilities in predicting CRIS-C patients responsive to Cetuximab pharmacological treatment, with adjustable sensitivity and specificity depending on the assignments of CRIS-C subtype secondary samples.

These results confirm that tumors harboring multiple CRIS phenotypes also express clinical features that reflect their multiple assignments. However, our study was limited to broad multi-label analysis. Dedicated studies with a higher number of multi-label samples may allow, in the future, to explore relations between different classes and how they affect the course of the disease.

## Conclusions

In summary, this work provides the biological and methodological basis for multi-label fuzzy classification of CRC. The results presented here confirm that capturing intratumor heterogeneity provides a more comprehensive picture of the biological and clinical features of human tumors. This information represents a milestone for deciphering tumor biology and for the development of novel therapeutic strategies, and it could be extended to other tumor types.

### Supplementary Information

**Additional file 1: Table S1.** Gene expression profile measured as reads count per genes of PDX collectionaccording to GeneCode version 27 quantified by featureCounts. Reads mapped to Mus Musculus genome were removed from the gene quantification.

**Additional file 2: Table S2.** Assignment of 620 TCGA samples using the Nearest Template PredictionmultiCRIS classifier. In the results, we identified samples with single label class CRIS, multi-label classes CRIS, and not classified. For classassignmentby NTP, FDR < 0.2 was considered significant.

**Additional file 3: Table S3.** Numbers and percentage of CRIS call assignments with different BH.FDR thresholds.

**Additional file 4: Table S4.** NTP-based multiCRIS classification of the RNA-seq bulk profiles of TCGA samples. Summary table of results with the number of samples with single-label class CRIS, multi-label class CRIS, and not primary assignment, primary assignment and any assignment.

**Additional file 5: Table S5.** Average of distance to the centroids of CRIS classes for primary and not primary assignments. The statistical significance has been computed by Student's T-test.

**Additional file 6: Table S6.** Overlap between CRIS classes in NTP multilabel assignments. Analysis performed on TCGA dataset.

**Additional file 7: Table S7.** Summary of the number of samples assigned to secondary classes with peculiar molecular features as MSI status, BRAF/KRAS mutation and EMT, WNT and paneth scoring. For dichotomous variables, hypergeometric p-value has been computed to assign statistical significance at the ratio Observed over the Expected. For continuous variable, Student's T-test was performed. NC, not assigned to reference class, i.e. not assigned to that class.

**Additional file 8: Figure S1.** Distribution of genes detected in scRNA-seq data of patient derived organoidswith a coverage of 1 to 5 reads for transcripts. **Figure S2.** Distribution of Pearson correlation values obtained by analysing unmatchedand matchedCRC PDO bulk / PDO pseudo-bulk pairs. **Figure S3.** CRIS classification of CRC single sample data from GSE132465, pseudo-bulk, and at scRNA-seq resolution. B) represents the distribution of CRIS distances evaluated on each single cell, grey points. Black points represent distances for cells with significant assignment. Red dots, represent the class distances on pseudo-bulks. C) waterfall of CRIS distances for significant assigned cells. **Supplementary Fig. 4.** Distribution of the CRIS classes on testing samples of TCGA and PDX datasets, using LSVM single-labeland adapted to multi-label. The TCGA dataset has 169 samples, while the PDX dataset 550 samples. **Figure S5.** Forest plots for LSVM-based models. Along y-axis, an identifier number for each test is reported. For each test, the odds ratioand its confidence interval are shown; the numeric label annoated on each odds ratio represents the p-value. Other details of the testsare listed below the corresponding plot.

**Additional file 9: Table S8a.** Counts data of pseudo-bulk RNA-seq profiles of CRC organoids. Pseudo-bulk profiles have been computed by summing the gene counts of scRNA-seq profiles. **Table S8b.** Counts data of bulk RNA-seq profiles of CRC organoids.

**Additional file 10: Table S9.** NTP-based multiCRIS classification in the bulk CRC organoids. NTP analysis has been performed on bulk PDO data identifying sample with Single-label CRIS classification, Multi-labels CRIS classification, and Not classification. For classassignmentby NTP, FDR < 0.2 was considered significant.

**Additional file 11: Table S10a.** NTP-based multiCRIS classification on scRNA-seq profiles of CRC organoids. NTP analysis has been performed on scRNA-seq PDO data identifying cell with single-label class CRIS, Multi-label classes CRIS, and not classification. For classassignmentby NTP, FDR < 0.2 was considered significant. **Table S10b.** NTP-based multiCRIS classification in the CRC organoids. Summary table of NTP results on total single-cell datasets and at sample levels with percentages of cells assigned, not classified, single-label CRIS class assignment and multi-labels CRIS assignments.

**Additional file 12: Table S11.** NTP-based multiCRIS classification on pseudo-bulk CRC patients. NTP analysis has been performed on CRC patients data identifying sample with Single-label CRIS classification, Multi-labels CRIS classification, and Not classification. For classassignmentby NTP, FDR < 0.2 was considered significant.

**Additional file 13: Table S12a.** NTP-based multiCRIS classification on the single-cell RNA-seq profiles from CRC patient. NTP analysis has been performed on scRNA-seq CRC patient data identifying cell with Single-label class CRIS, Multi-labels classes CRIS, and Not classification. For classassignmentby NTP, FDR < 0.2 was considered significant. **Table S12b.** NTP-based multiCRIS classification on the single-cell RNA-seq profiles from CRC patient. Summary table of NTP results on total single-cells datasets and at sample levels with percentages of Assigned, Not Classified, Single-label CRIS class assignment and Multi-labels CRIS assignments.

**Additional file 14: Table S13.** Single-label performance evaluations of the considered algorithms on testing data, comparing their results with the ones of the original NTP approach.

**Additional file 15: Table S14.** Multi-label performance evaluations of the considered algorithm-adapted strategies on testing data, comparing their results with multi-label assignments obtained from the multiCRIS version of the NTP approach.

**Additional file 16: Table S15.** Multi-label assignments of the algorithm-adapted LSVM on TCGA testing samples, compared with multi-label assignments from the multiCRIS version of the NTP approach.

**Additional file 17: Table S16.** Multi-label assignments of the algorithm-adapted LSVM on PDX samples, compared with multi-label assignments from the multiCRIS version of the NTP approach.

**Additional file 18: Table S17.** Genes selected based on feature importance.

**Additional file 19. **Supplementary material of "Multi-label transcriptional classification of colorectal cancer reflects tumour cell population heterogeneity.

## Data Availability

• All data generated during this study are included in this published article. Raw data are available at EGAS00001006492 (https://ega-archive.org/studies/EGAS00001006492, Bank of metastatic colorectal cancer (mCRC) of Patient-Derived Xenografts (PDXs), RNA-seq) [58], EGAS00001006214 (https://ega-archive.org/studies/EGAS00001006214, scRNA of five mCRC organoids in basal conditions, scRNA-seq) [59], and EGAS00001007051 (https://ega-archive.org/studies/EGAS00001007051, Bank of primary sites (PRs) colorectal cancer of Patient-Derived Xenografts (PDXs), RNA-seq) [60]. • The dataset of scRNA-seq derived from human tumor analyzed during the current study are available in the GEO repository, GSE132465 (https://www.ncbi.nlm.nih.gov/geo/query/acc.cgi?acc=GSE132465) [31].

## Abbreviations

CRC: ColoRectal cancer
CRIS: ColoRectal Cancer Intrinsic Subtype
CMS: Consensus molecular subtype
PDX: Patient-derived xenografts
MSI: Microsatellite instable
mla: Multi-label adapted
LSVM: Linear Support Vector Machine
PSVM: Polynomial Support Vector Machine
RF: Random Forest
