# Supplementary material for: Multi-label transcriptional classification of colorectal cancer reflects tumor cell population heterogeneity
Source: Genome Med. 2023 May 15;15:37. doi: 10.1186/s13073-023-01176-5 (PMC10184353; doi:10.1186/s13073-023-01176-5)
Supplement: Supplementary file 8 — Additional file 8: Figure S1. Distribution of genes detected in scRNA-seq data of patient derived organoidswith a coverage of 1 to 5 reads for transcripts. Figure S2. Distribution of Pearson correlation values obtained by analysing unmatchedand matchedCRC PDO bulk / PDO pseudo-bulk pairs. Figure S3. CRIS classification of CRC single sample data from GSE132465, pseudo-bulk, and at scRNA-seq resolution. B) represents the distribution of CRIS distances evaluated on each single cell, grey points. Black points represent distances for cells with significant assignment. Red dots, represent the class distances on pseudo-bulks. C) waterfall of CRIS distances for significant assigned cells. Supplementary Fig. 4. Distribution of the CRIS classes on testing samples of TCGA and PDX datasets, using LSVM single-labeland adapted to multi-label. The TCGA dataset has 169 samples, while the PDX dataset 550 samples. Figure S5. Forest plots for LSVM-based models. Along y-axis, an identifier number for each test is reported. For each test, the odds ratioand its confidence interval are shown; the numeric label annoated on each odds ratio represents the p-value. Other details of the testsare listed below the corresponding plot. [file 13073_2023_1176_MOESM8_ESM.docx]

**Supplementary figures**


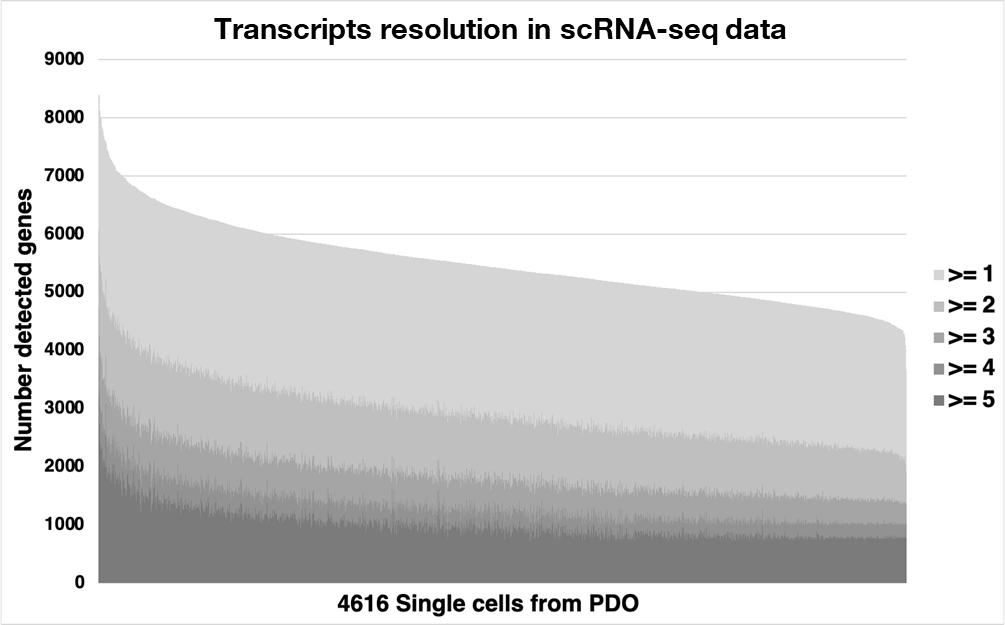


**Figure S1: Distribution of genes detected in scRNA-seq data of patient derived organoids (PDO) with a coverage of 1 to 5 reads for transcripts.**

**
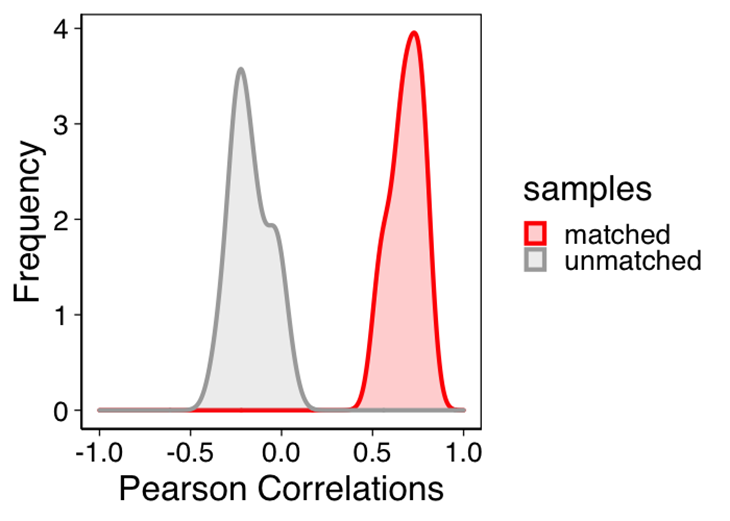
**

**Figure S2: Distribution of Pearson correlation values obtained by analysing unmatched (grey line) and matched (red line) CRC PDO bulk / PDO pseudo-bulk pairs (Student’s T-test; p-value < 2.993e-07).**

­­­­­­­­­­­­­­­­­­**
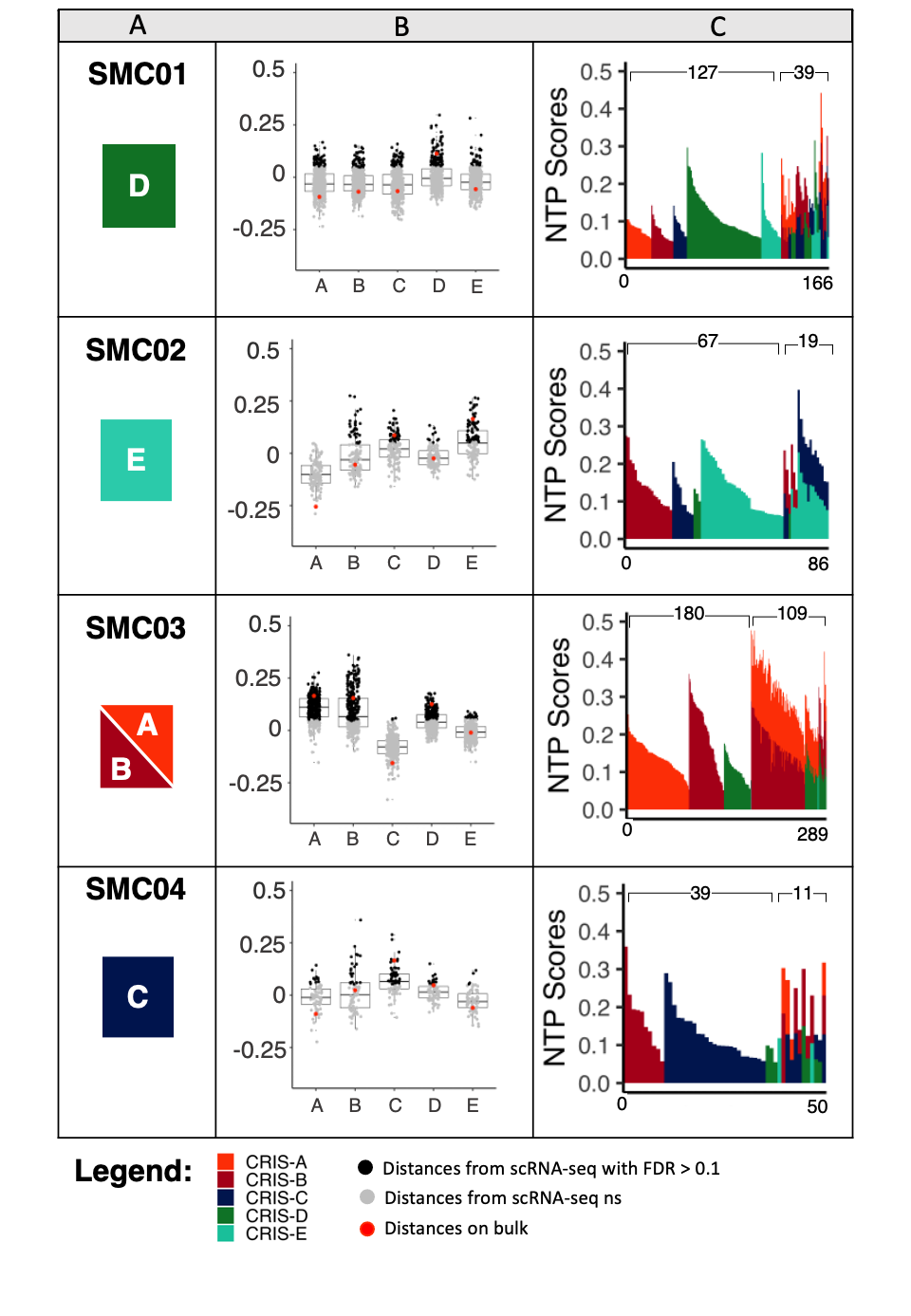
**

**Supplementary Figure 3 Part 1**

**
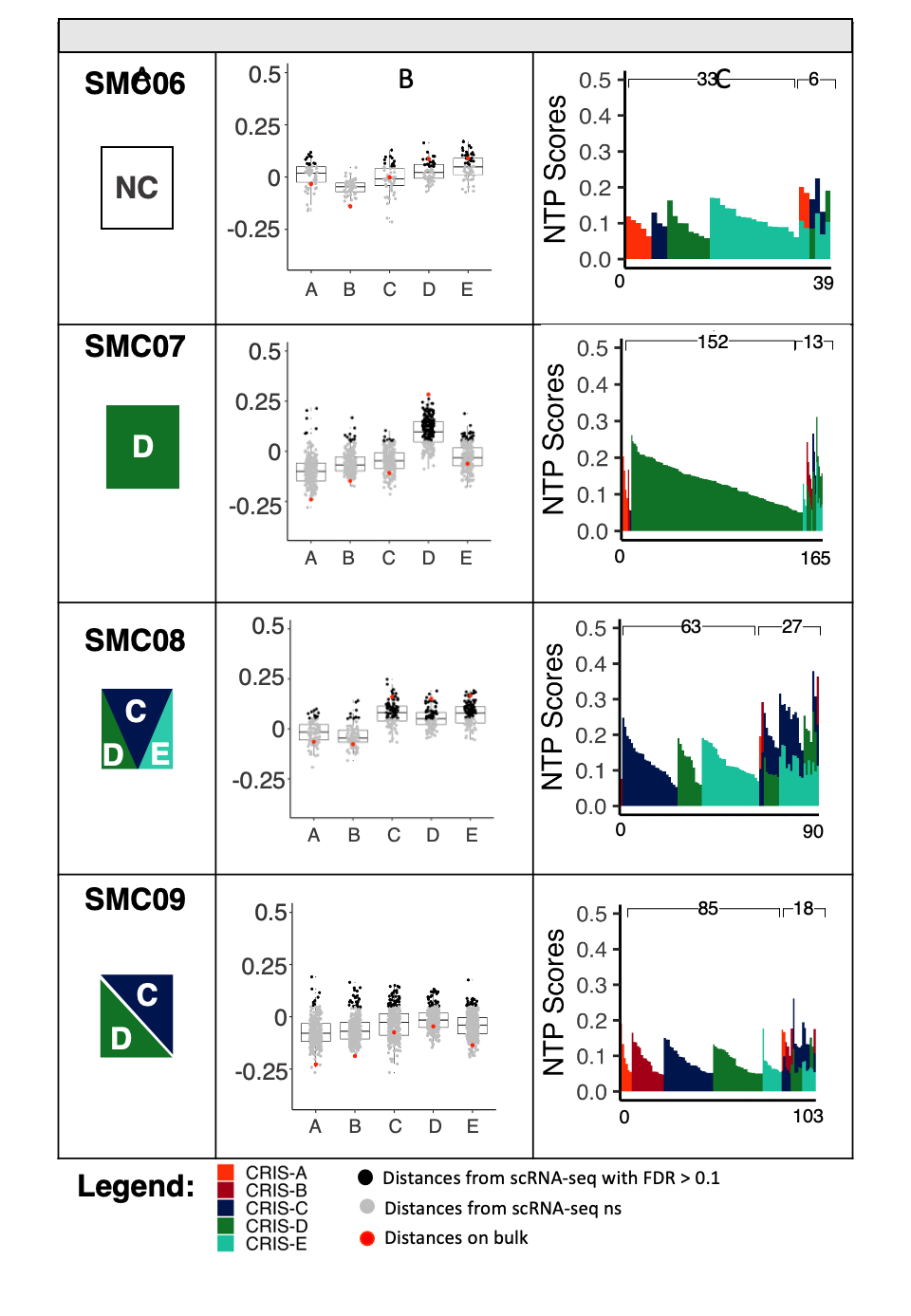
**

**Supplementary Figure 3 Part 2**

**
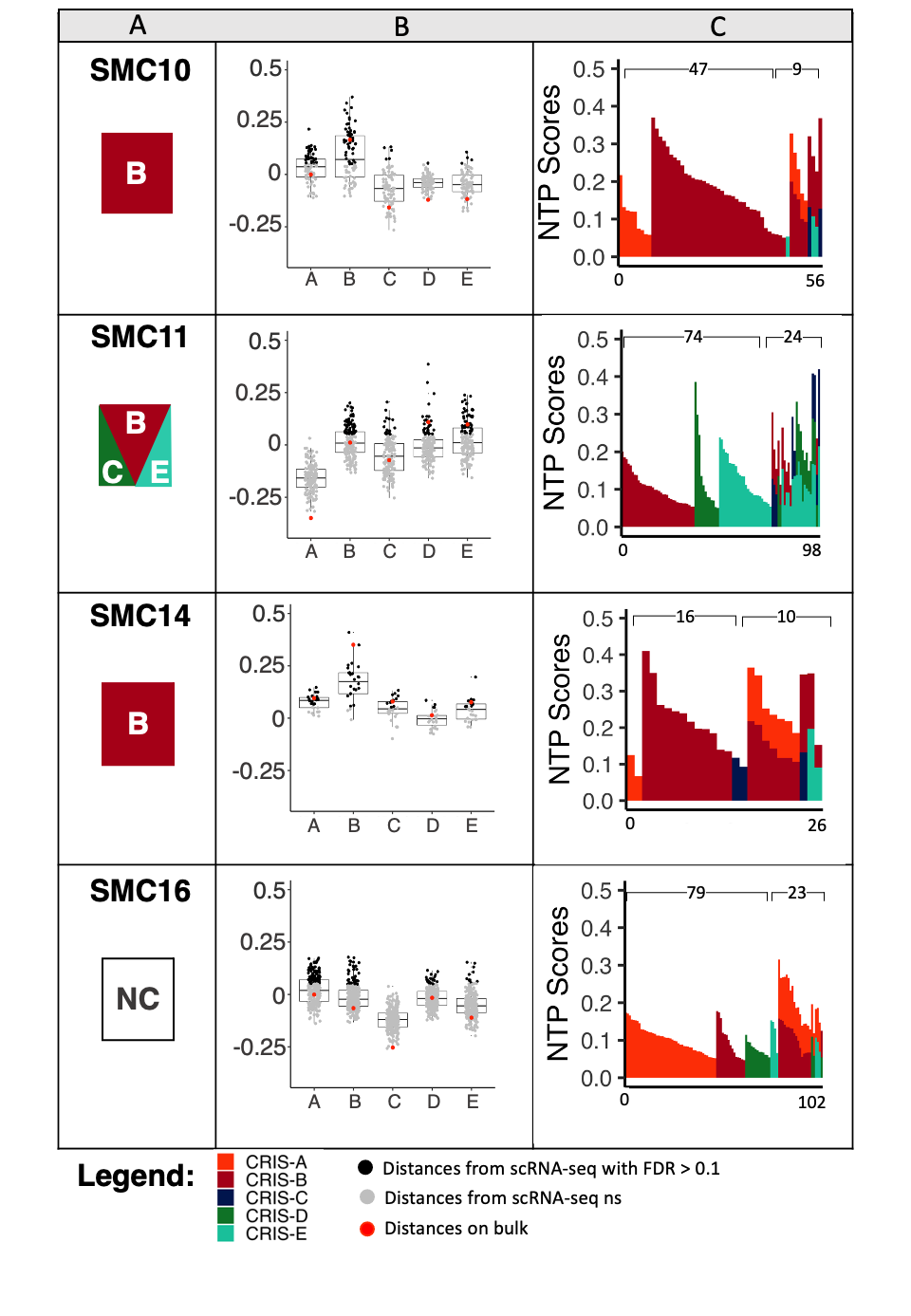
**

**Supplementary Figure 3 Part 3**

**
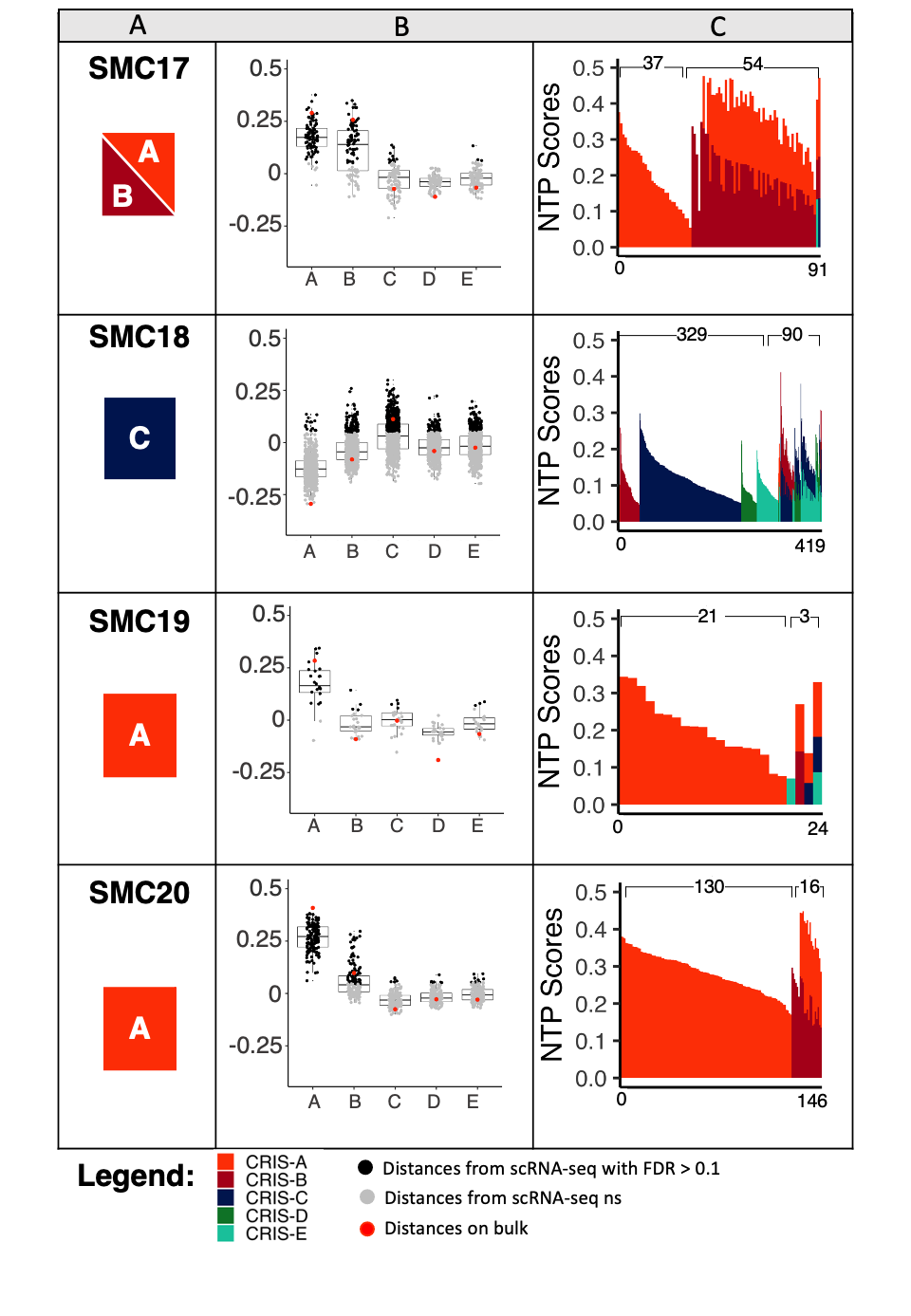
**

**Supplementary Figure 3 Part 4**

**
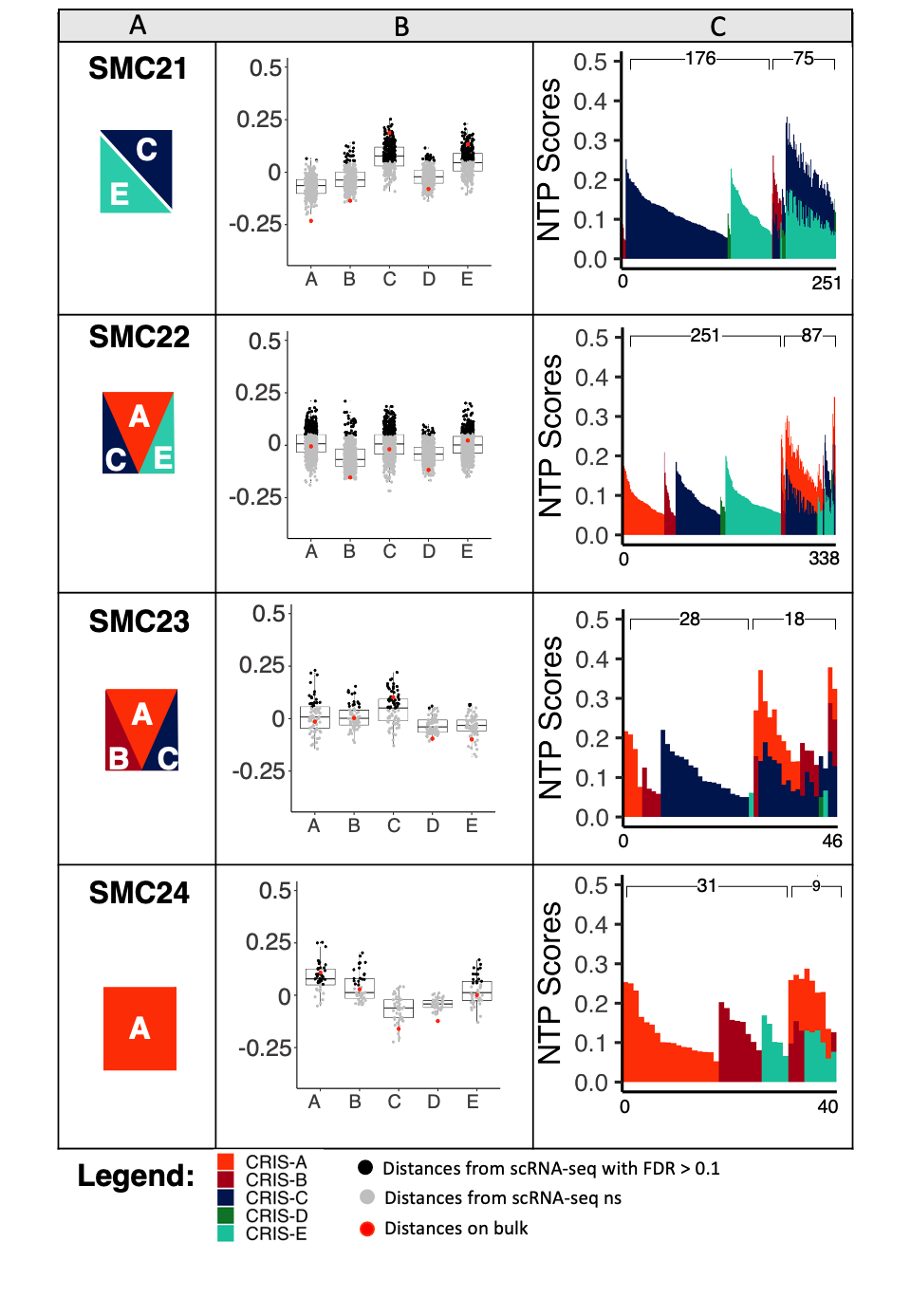
**

**Supplementary Figure 3 Part 5**

**
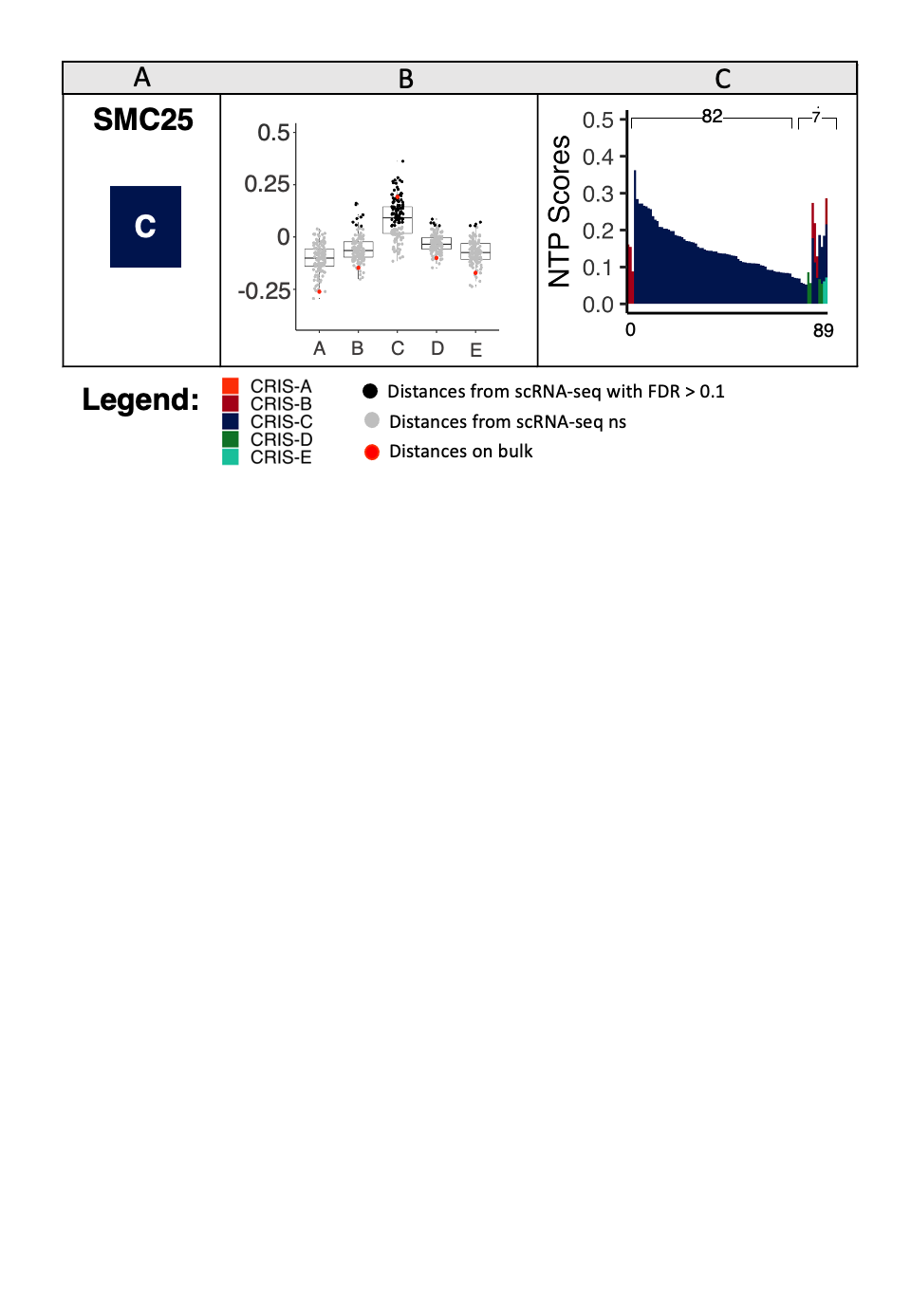
**

**Supplementary Figure 3 Part 6**

**Figure S3: CRIS classification of CRC single sample data from GSE132465, pseudo-bulk (Column A), and at scRNA-seq resolution (B and C). B) represents the distribution of CRIS distances evaluated on each single cell, grey points. Black points represent distances for cells with significant assignment. Red dots, represent the class distances on pseudo-bulks. C) waterfall of CRIS distances for significant assigned cells.**

**
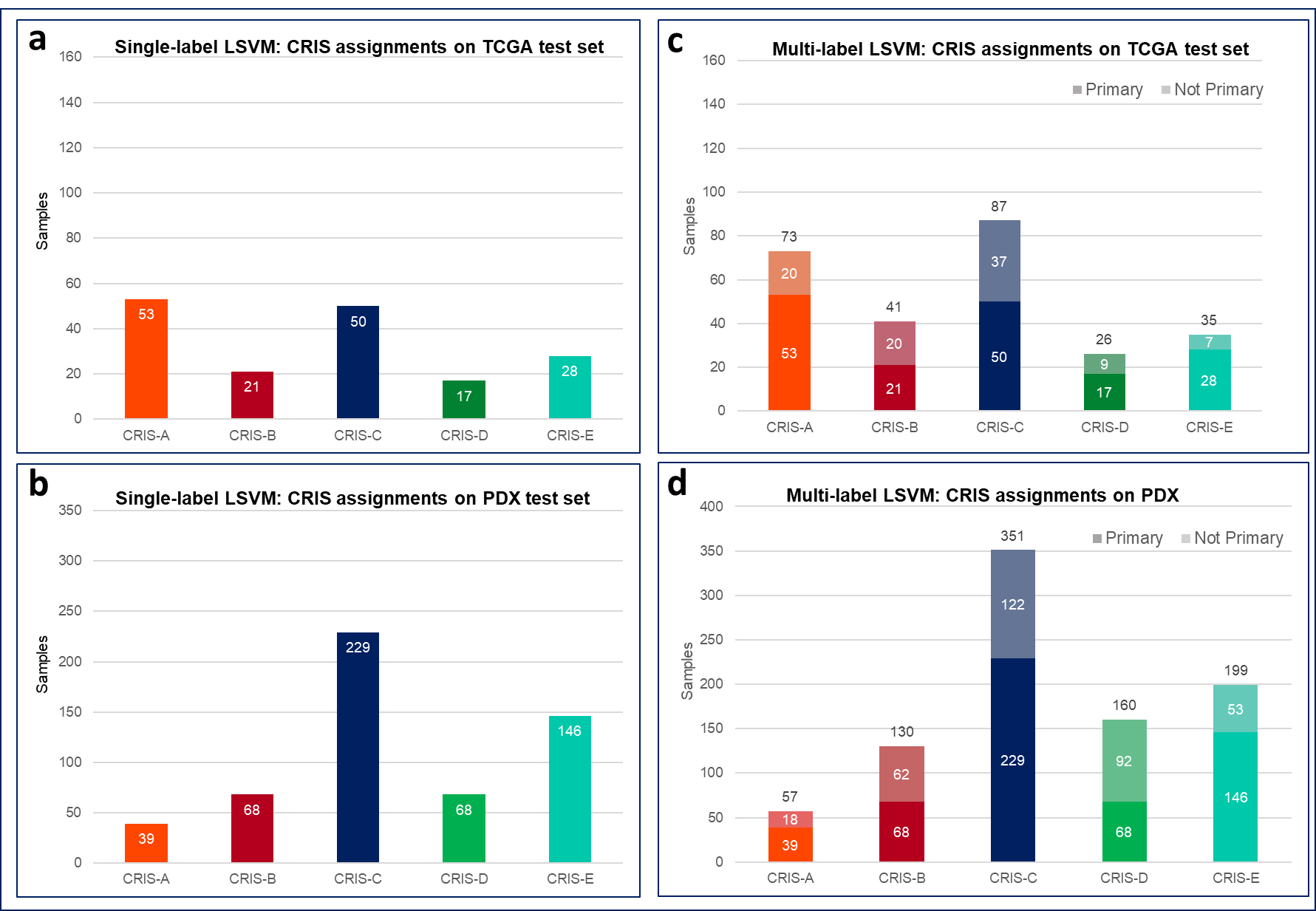
**

**Supplementary Figure 4. Distribution of the CRIS classes on testing samples of TCGA and PDX datasets, using LSVM single-label (a, b) and adapted to multi-label (c, d). The TCGA dataset has 169 samples, while the PDX dataset 550 samples.**

**
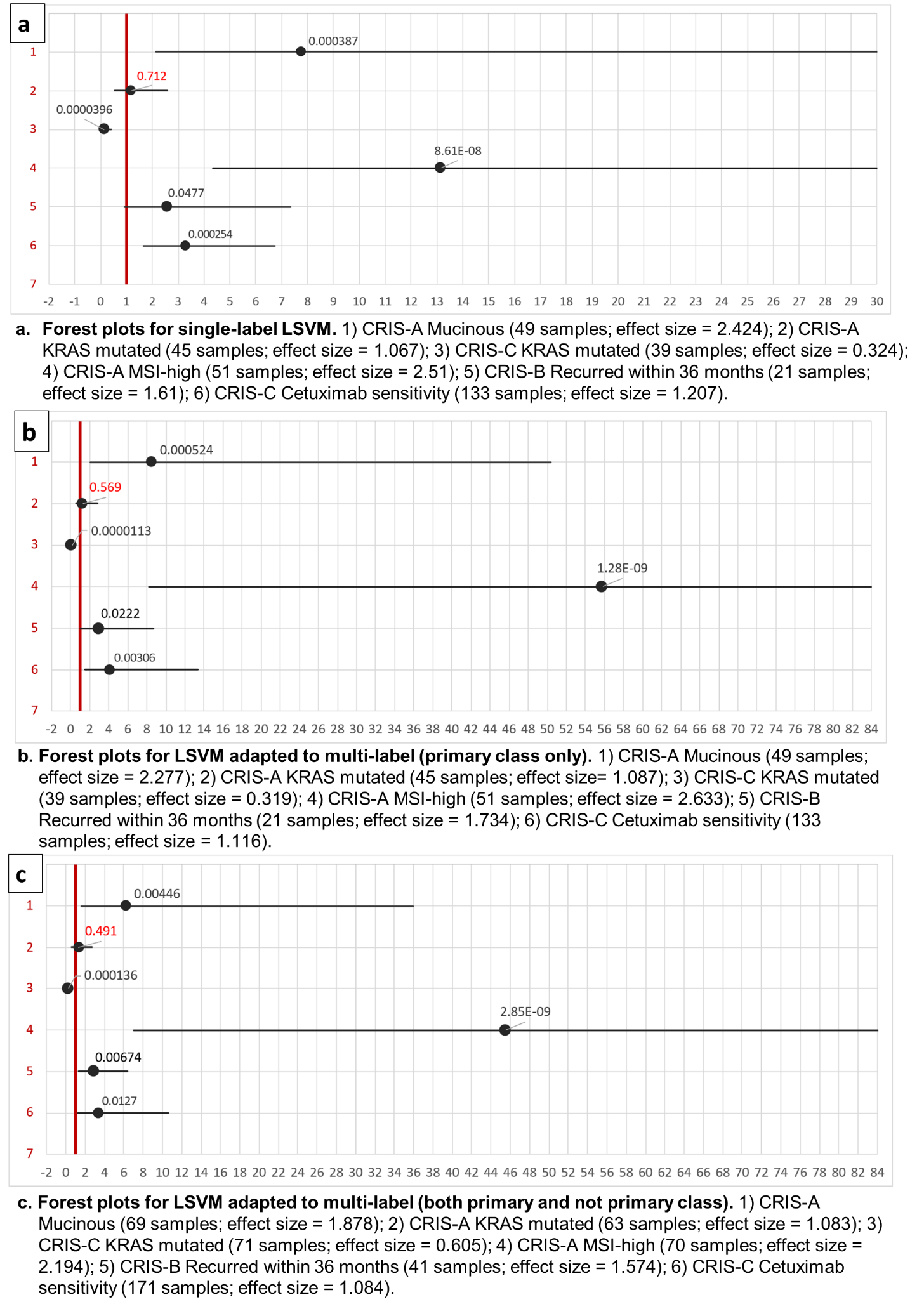
**

**Figure S5. Forest plots for LSVM-based models. Along y-axis, an identifier number for each test is reported. For each test, the odds ratio (represented as a full point) and its confidence interval are shown; the numeric label annoated on each odds ratio represents the p-value (coloured in red when not significant). Other details of the tests (number of class samples and effect size) are listed below the corresponding plot.**
