# Supplementary material for: Multi-label transcriptional classification of colorectal cancer reflects tumor cell population heterogeneity
Source: Genome Med. 2023 May 15;15:37. doi: 10.1186/s13073-023-01176-5 (PMC10184353; doi:10.1186/s13073-023-01176-5)
Supplement: Supplementary file 19 — Additional file 19. Supplementary material of "Multi-label transcriptional classification of colorectal cancer reflects tumour cell population heterogeneity. [file 13073_2023_1176_MOESM19_ESM.pdf]

Supplementary material of "Multi-label transcriptional classification of colorectal cancer reflects tumour cell population heterogeneity"

Silvia Cascianelli[1,*], Chiara Barbera[1,*], Alexandra Ambra Ulla[2,*], Elena Grassi[2,3], Barbara Lupo[2,3], Diego Pasini[4,5], Andrea Bertotti[2,3], Livio Trusolino[2,3], Enzo Medico[2,3,+], Claudio Isella[2,3,∮,+], Marco Masseroli[1,∮,+]

[1]*Department of Electronics Information and Bioengineering, Politecnico di Milano, Piazza Leonardo da Vinci 32, 20133, Italy*
[2]*Department of Oncology, University of Torino, Candiolo (TO), S.P. 142, km 3.95, 10060, Italy*
[3]*Candiolo Cancer Institute, FPO-IRCCS, Candiolo (TO), S.P. 142, km 3.95, 10060, Italy*
[4]*IEO, European Institute of Oncology IRCCS, Department of Experimental Oncology, Via Adamello 16, 20139 Milan, Italy*
[5]*University of Milan, Via A. di Rudini 8, Department of Health Sciences, 20142 Milan, Italy*

* These authors contributed equally to the work
\+ These authors jointly supervised the work
∮ Corresponding authors: marco.masseroli@polimi.it, c.isella@unito.it

1 Datasets

- **TCGA dataset**: RNA-seq bulk expression data from the Colon adenocarcinoma (COAD) and Rectum adenocarcinoma (READ) projects of The Cancer Genome Atlas (TCGA)[1] were pre-processed preserving 620 primary tumoral samples and normalizing the expression values in CPM (counts per million)[2] using the CPM function of the edgeR package[3].
- **PDX dataset**: RNA-seq bulk profiles from 646 liver metastatic colorectal cancer samples of patient derived xenografts (PDXs)[4] were generated, and then pre-processed preserving 606 samples, whose expression values are normalized in CPM.
- **scRNA-seq GSE132465 dataset**: This single-cell RNA-seq dataset from the NCBI Gene Expression Omnibus database[5] was pre-processed and 4,291 epithelial colorectal cancer cells were selected.
- **PDO datasets**: Both scRNA-seq and bulk profiles were generated for an in-house collection of 5 CRC patient-derived organoids (EGAS00001006214, PDO), whose expression values are normalized in CPM, after pre-processing.
- **Pseudo-bulk profiles** were generated from GSE132465 and PDO scRNA-seq datasets, and then normalized in CPM values.

2 CRIS classification using NTP and its multiCRIS evolution

2.1 CRIS signature and original NTP single-label classification

The CRIS signature is a set of 565 genes, listed in Supplementary Data 8 of Isella *et al.*[6], defined for colorectal cancer data aligned to the hg19 reference human genome. Since our datasets are aligned to the newer GRCh38 reference genome, some of the CRIS genes appear with different aliases. In order to solve the conversion problem and be able to use the original NTP classifier[6], we identified the correspondences based on the Entrez Gene ID[7] of the genes and created conversion tables; a single pseudo-gene (LOC645166) was removed, as missing in GRCh38 data. Thus, in this work the used *NTP signature* refers to the set of remaining 564 CRIS genes still available in the GRCh38 reference genome.

The NTP single-label classification algorithm is the original Nearest Template Prediction approach developed by Isella *et al.*[6], here applied always considering all remaining 564 genes of the NTP signature as features of interest. By construction, this algorithm cannot classify each sample individually. In fact, only after an intra-dataset data normalization (computing Z-score values of each sample), NTP computes the distances of each normalized sample profile from the template centroids (Supplementary Data 8 of Isella *et al.*[6]) of each CRIS class. Then, NTP assigns a sample only to the class at the minimum confident distance, considering as threshold for sample confident distance its Benjamini–Hochberg false discovery rate (BH.FDR) < 0.2, as previously reported[6].

In this work, we obtained NTP single-label assignments using 100,000 random gene resamplings for the computation of the Benjamini–Hochberg false discovery rate values, representing the confidence associated with each distance between a sample expression profile and each of the CRIS class centroids. From these classifications, we then extracted only subsets of confidently classified samples, i.e., samples where the BH.FDR of the assigned class distance is lower than 0.2; precisely, we kept 562 samples for TCGA and 550 samples for PDX datasets.

2.2 CRIS classification

After preprocessing, reference classifications of TCGA and PDX data were obtained using the original NTP CRIS classification method. NTP classification was performed also on pseudo-bulk/bulk/scRNA-seq data of PDO samples and on pseudo-bulk/scRNA-seq data of CRC patients (GSE132465)[5]. For single-cell data, all genes with at least one read per gene in a sample were included, for a total of 95% of the CRIS genes.

2.3 MultiCRIS: NTP multi-label classification

To deal with a multi-label context, we adapted the NTP method by computing class-specific thresholds and comparing such thresholds with the distances of a sample from each NTP class centroid; this allows assigning a sample i also to additional classes j, if their calls are confident ($BH.FDR_{i,j} < 0.2$). The thresholds are computed using the following procedure, separately on the sets of confident single-label classified TCGA and PDX samples. For each class, first we computed the distribution of the NTP correlation scores $c_{i,j} = 1 - d_{i,j}$, where i is a sample confidently classified to its class j and $d_{i,j}$ is its NTP distance from the centroid of the assigned class j; the score distributions of each class for the TCGA dataset are reported in Figure 1, while the same distributions for the PDX dataset are reported in Figure 2. Then, we computed the 5th percentile of the NTP correlation scores of each class j in a dataset and took this value as the dataset class threshold t_j, then used to decide the multi-label assignment of a sample to the class j. The choice of a 5th percentile threshold is motivated by the aim of excluding the smallest, albeit confident, NTP correlation scores from multi-label assignments of inherently heterogeneous samples: indeed, when a score is confident but below the class-specific threshold the class is not assigned to the sample under exam.

After computing for each dataset its class thresholds t_j, we transformed its single-label targets into multi-label targets, to be used in the subsequent multi-label classification testing. Each multi-label target is a binary vector that can host multiple class assignments for a sample (e.g., the binary vector 10001 means that the sample is assigned to CRIS-A and CRIS-E classes).

This procedure confirms the primary assignment of the NTP single-label, and possibly assigns secondary classes that must be confident calls but also exceed the minimum accepted membership score. The original CRIS class assigned by the single-label NTP is referred to as the sample primary class. For each sample i, its corresponding class binary vector cl_i is built as follows:

- If sample i is confidently assigned to primary class j (i.e., if $BH.FDR_{i,j} < 0.2$), then set $cl_{i,j} = 1$;
- If class j is not the primary class of sample i, but its score is $c_{i,j} >= t_j$ and its BH.FDR is $BH.FDR_{i,j} < 0.2$, then set $cl_{i,j} = 1$;
- In all other cases, set $cl_{i,j} = 0$.

Thus, if a sample has a NTP class correlation score greater than the class-specific threshold, the NTP multi-label classification (multiCRIS) assigns the sample to that class, as long as this assignment occurs with confidence (i.e., with a BH.FDR < 0.2). So-obtained multiCRIS calls were then used as reference targets for testing the here developed single-sample multi-label models.

3 Single-sample classification models

3.1 Feature space, supervised targets and models under exam

For the implementation of single-sample CRIS classifiers, we used only confident (i.e., with BH.FDR < 0.2) NTP single-label assignments as target references for the training and testing phases, for a total of 562 confidently classified TCGA samples and 550 confidently classified PDX samples. For our feature space of interest, we selected the 564 genes belonging to the original CRIS signature, identified as described in the previous section 2.1. This choice allowed focusing on a set of genes proved to be relevant for the CRIS subtyping task, other than making a fairer comparison with pre-existing approaches. Furthermore, this feature dimension, comparable with the number of samples under analysis, allowed avoiding the curse of dimensionality and contributed to mitigate the overfitting risk. To build our classification models we considered and benchmarked the following algorithms, which are a representative set of the ones typically used in the field:

- Support Vector Machines (SVM), with either linear (LSVM), polynomial (PSVM), or Gaussian radial basis function (GRBF-SVM) kernel: they use a subset of samples (called support vectors) to compute a boundary between two classes in a binary classification setting. Since our research considers a multi-class settings (we have 5 CRIS classes that can be assigned), each SVM classifier is actually made of 5 binary SVMs that compute a boundary for each CRIS class in a one-vs-all approach. Then, a softmax transformation layer is used to assign the class with the highest transformed class membership.
- Random Forest (RF): an ensemble method that uses bootstrap aggregation (i.e., bagging) of several binary decision trees trained independently on random subsets of features to assign the most likely single CRIS class to each sample.

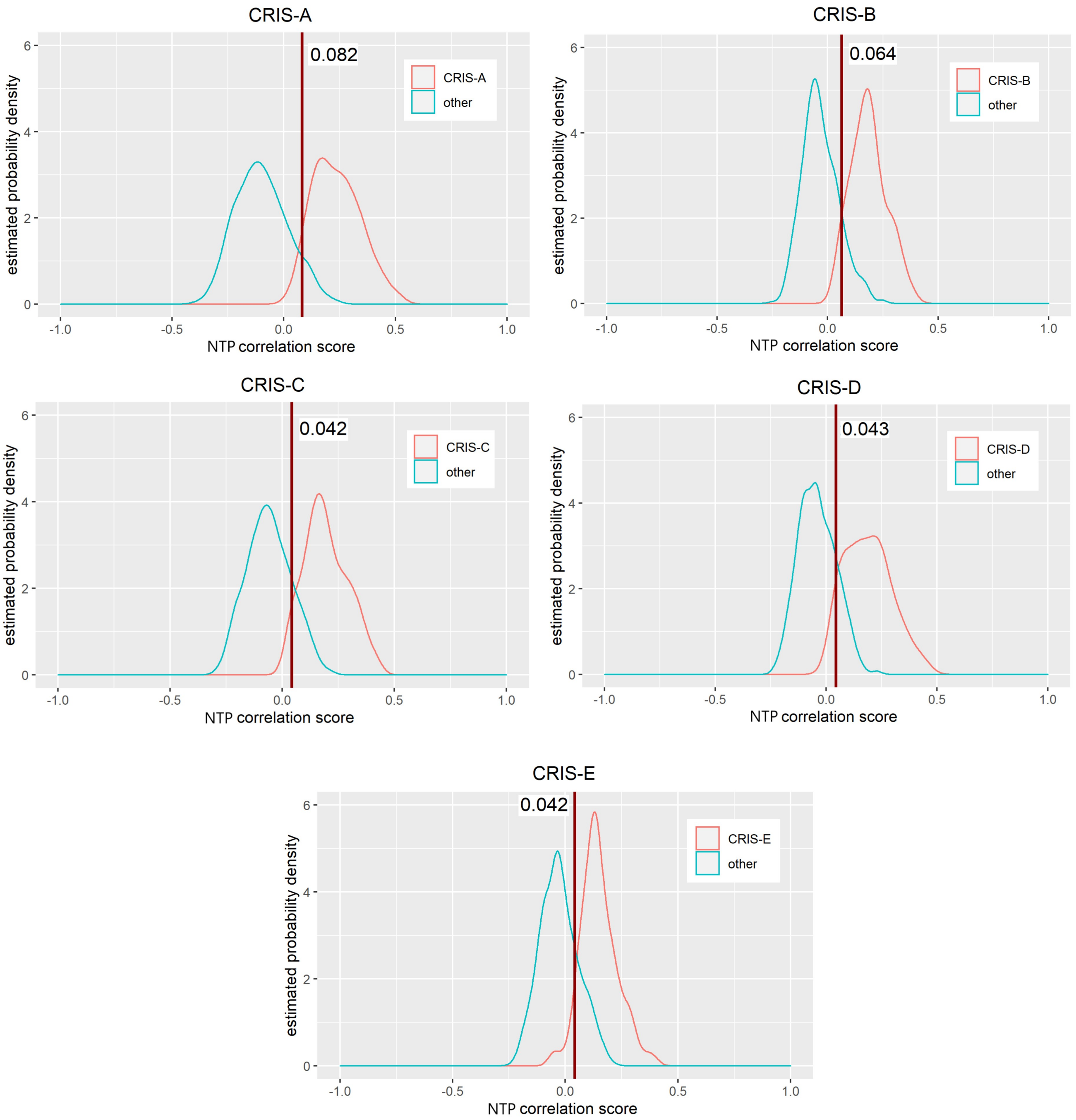

Figure 1. Distribution of NTP correlation scores of TCGA confident samples for each CRIS class. On the x-axis the NTP correlation score is displayed. The orange curve refers to samples assigned to the considered class, the blue curve refers to samples not belonging to the considered class. The red line represents the class-specific threshold.

- Extreme Gradient Boosting (XGBoost): another ensemble method that performs boosting aggregation, i.e., builds and adds one tree at a time, introducing such a new weak learner to improve shortcoming of existing trees by assigning more weight on instances with wrong predictions and high errors.
- Neural Network (NN): a fully connected Feed-Forward Neural Network with a single hidden layer and sigmoid activation functions.

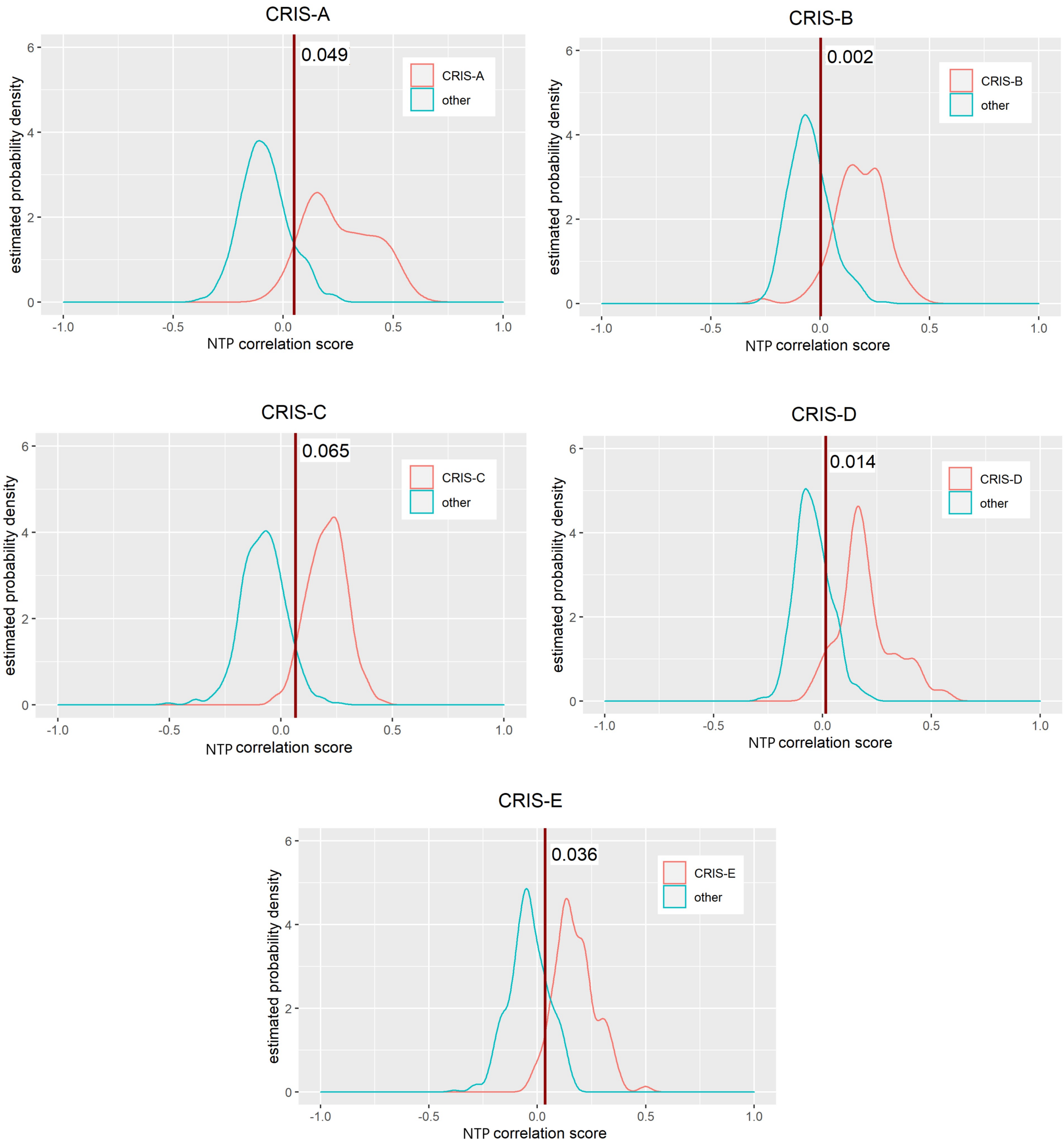

Figure 2. Distribution of NTP correlation scores of PDX confident samples for each CRIS class. On the x-axis the NTP correlation score is displayed. The orange curve refers to samples assigned to the considered class; the blue curve refers to samples not belonging to the considered class. The red line represents the class-specific threshold.

To create the classification models we used the *caret* R package[8], which provides a unified way of training and testing classification algorithms. In turn, *caret* uses the *e1071*[9] and *kernlab*[10]packages for modelling and working on SVMs, the *randomForest*[11] package for the namesake classifier, and xgbTree[12] for the XGBoost ensemble.

Additionally, we compared the results of such models with the performances of the Top Scoring Pairs algorithm[6], the only already existing attempt of a single-sample CRIS classifier; this was previously obtained from the original NTP classifier. The

TSP approach predicts the CRIS class of a sample by aggregating the votes of 5 rule-based binary classifiers (one for each class). It is trained to recognize CRIS classes by defining decision rules based on the expression levels of pairs of genes of the CRIS signature; specifically, a gene pair is chosen if characterized by inverted relative expressions in two different CRIS classes. For example, if gene X has a higher expression than gene Y in CRIS-A and, conversely, a lower expression than gene Y in CRIS-B, then such pair of genes (X and Y) can be used to derive a classification rule that contributes in distinguishing the class CRIS-A from the CRIS-B.

3.2 Training and testing data splitting to develop single-sample classifiers

The splitting of TCGA data in training (70% - 393 samples) and testing (30% - 169 samples) data was performed maintaining the same class distribution of the NTP single-label classification, both in the training and test sets. The NTP single-label class distributions (fully coincident with the NTP multiCRIS primary class distributions) are reported in Table 1, together with the one of the testing PDX dataset.

DATA SETS	PRIMARY CRIS-A	PRIMARY CRIS-B	PRIMARY CRIS-C	PRIMARY CRIS-D	PRIMARY CRIS-E	TOTAL SAMPLES
**TCGA ALL**	150	69	155	86	102	562
**TCGA TRAIN**	106	47	110	59	71	393
**TCGA TEST**	44	22	45	27	31	169
**TCGA %**	27%	12%	28%	15%	18%	100%
**PDX**	111	82	169	76	112	550
**PDX %**	20%	15%	31%	14%	20%	100%

Table 1. CRIS class distributions based on the target NTP labels (i.e., NTP single-label classes fully coincident with the NTP multiCRIS primary classes) for both TCGA and PDX data sets. Notice that CRIS-A class is under-represented in PDX vs. TCGA data as a consequence of the scarcity of MSI cases in PDX data.

Moreover, we kept the same proportions also for cancer type (Colon, Mucinous and Rectal adenocarcinoma) and MSI status. Finally, since the disease-free annotations (status and months up to the last follow-up) are particularly interesting for prognostic validation, we paid attention at:

- Including all samples with missing information on disease-free status and/or disease-free months in the training set;
- Including all samples with recurrences after 5 years (60 months) in the training set, as they are not particularly relevant for the time horizon considered in our final survival analyses at medium-term;
- Including in the testing set most of the prognostically relevant samples, i.e., annotated as disease-free, for more than 60 months, or recurred within the first 60 months, since the latter ones are essential for the validation of poor prognosis;
- Dividing between training and testing set the samples censored before 60 months.

During the training phase, we used a stratified 10-fold cross-validation approach (in which each of the 10 folds had the same balancing of primary classes as in the entire training set) together with a grid search to perform hyper-parameter tuning and optimize the performance of the considered classifiers. We examined appropriate ranges for all the hyperparameters of the algorithms to be tuned, including the misclassification penalty of any SVM, since too high values can increase overfitting risk. The so found best hyper-parameter values were then used to train each model one last time on the entire training set. After last training on the entire TCGA training set and testing on the TCGA test set left aside, the PDX dataset was used only to further test the performances of all the considered classifiers on completely independent data. The combined use of 10-fold cross-validation and hold-out test set from TCGA, together with the additional step of independent testing on PDX was meant to further face overfitting risk and focus on models providing both good and robust performance. Additionally, to further evaluate our models regarding the overfitting risk for the limited sample size, we compared both global accuracy and local (class specific) results on testing when using either 10-fold or 5-fold cross-validation; no appreciable differences were found (see Supplementary Table 18).

3.3 Single-sample multi-label classification models from algorithm adaptation strategy

We developed an algorithm-adaptation strategy to derive multi-label classifiers directly from the single-sample classification methods used in the single-label setting (similarly to what done to derive the multi-label NTP classifier - multiCRIS - from its original single-label version). Thus, our multi-label approaches are not trained based on multiCRIS assigments, but just based

on primary NTP classes like their single-label versions; multiCRIS assigments were then simply used as references on the testing samples to compute all the performance measures of interest during the test phases.

Each of our single-sample classification models (LSVM, PSVM, GRBF-SVM, RF, XGBT and NN) computes for each of its training samples a CRIS score for every class, before assigning the sample to its primary class through a softmax score transformation. Thus, in order to possibly assign one or more additional classes to a sample based on its CRIS class scores, for each of these classifiers we computed a class-specific CRIS score threshold for each of the five CRIS classes through the following approach. First, we extracted the class scores returned by the single-label classifier without using its final softmax layer, as to move towards the multi-label context. Indeed, the softmax transformation at the end of a classification pipeline is meant to strengthen the algorithm in returning a single called class; conversely, the class scores computed before the softmax transformation represent how much a sample belongs to each CRIS class. Then, in our procedure the scores for each sample are independently normalized class-by-class to a $[0,1]$ range, using the min and max score values of each class in the TCGA training data. This normalization is used to obtain comparable class membership values for each sample. Always considering training samples only, we took the distributions of these normalized membership values grouped by class, and from each distribution we obtained a class-specific threshold as the class mean value diminished by 2.5 times the class coefficient of variation. By means of these thresholds, we are able to capture samples associated with the CRIS class under exam also when it is not the primary class, based on the multi-label assignments provided by the NTP multiCRIS classification.

If a testing sample has a normalized class score greater or equal to the corresponding class-specific threshold, the multi-label adapted algorithm assigns the sample to that class. Thus, using the class thresholds and min and max parameters for scaling computed on the training set (Table 2), multi-label adapted versions of the original single-label classifiers are made available to assign to any unseen testing sample not only the primary class, but possibly multi-label predictions. Such class-specific thresholds and scaling parameters are ready to be used on completely independent testing data; their creation method does not invalidate the purpose of obtaining single-sample classifiers, since all thresholds and scaling parameters are computed on training data, only during the threshold definition phase, and are then simply applied on testing data without further modifications. Classification performances obtained on unseen testing datasets of different types demonstrate the efficacy and reliability of this approach, as our reported results show.

CRIS class	CRIS-A	CRIS-B	CRIS-C	CRIS-D	CRIS-E
**Min class score for scaling**	-8.05103	-4.38325	-8.63258	-3.89980	-4.15411
**Max class score for scaling**	11.2631	7.11240	6.91121	12.2845	7.48886
**Threshold**	0.44553	0.47338	0.55210	0.32012	0.47956

Table 2. Min and max class score parameters for scaling and class thresholds, both computed on the training set, to be used on any individual sample in order to apply our algorithm adaptation to multi-label classification.

3.4 Performance metrics and target references for single-sample classifiers

For the evaluation of our single-sample single-label classifiers we used original NTP[6] assignments as target references and state-of-the-art classification metrics derived from the confusion matrices. In particular, we refer to TP_j as the True Positives for class j (samples of class j that are assigned to class j), to TN_j as the True Negatives for class j (samples not belonging to class j that are correctly not assigned to class j), to FP_j as the False Positives for class j (samples not belonging to class j that are wrongly assigned to class j) and to FN_j as the False Negatives for class j (samples belonging to class j that are wrongly not assigned to class j). We used the following metrics:

Accuracy: proportion of samples correctly classified. Given $k = 5$ CRIS classes, and N samples, the accuracy A is computed as:

$$A = \sum_{j=1}^{K} \frac{TP_j}{N} \tag{1}$$

Precision: computed for each class j, the precision represents the percentage of samples assigned to j that effectively belongs to j. Given the j-th CRIS class, the precision P_j for class j is computed as:

$$P_j = \frac{TP_j}{TP_j + FP_j} \tag{2}$$

A high value of precision represents the confidence with which each assignment to class j can be considered.

Recall: computed for each class j, the recall represents the percentage of samples originally belonging to class j that is assigned to j. Given the j-th CRIS class, the recall R_j for class j is computed as:

$$R_j = \frac{TP_j}{TP_j + FN_j} \tag{3}$$

A high value of recall represents the capability of the algorithm of recognizing the samples of class j.

Additionally, we computed the Areas under the Receiver Operating (AUROC) and Precision-Recall (AUPRC) curves. For the former, we used a well-established generalization of the AUROC for Multiple Classification[13], while for the AUPRC we used the mean average precision score (AP) formula with either weighted- (with respect to the size of each class), micro- (considering all the TPs of any class) or macro- (considering the average results in each class) averaging. AP computes the area under a precision-recall curve as the weighted mean of precisions achieved at different threshold, with the increase in recall from the previous threshold used as the weight. This is the most suitable choice to find the AUPRC since a linear interpolation of points on the precision-recall curve (to compute a trapezoidal area) would provide an overly-optimistic measure of classifier performance[14].

To evaluate our multi-label single-sample classifiers, we used as target references the multi-label calls obtained with the here introduced NTP multiCRIS approach. Yet, to consider just the most reliable secondary classes as targets, the NTP multiCRIS results were further compared to class-specific thresholds. These thresholds were computed for each class as the 5th percentile of the class NTP scores on the entire TCGA and PDX sets separately. By applying such thresholds on each testing sample we obtained more reliable targets against which our single-sample machine learning adapted classifiers could be tested: indeed, any confident assignment that still has an under-threshold class membership is discarded. Then, some of the metrics used for the evaluation of multi-label classifiers are comparable to the single-label ones, since the definition of True Positives, False Positives, True Negatives, False Negatives, Precision and Recall is analogous to the one reported above, even if based on multi-label target references. Also, we considered Y_i as the set of labels of the classes to which the sample i effectively belongs, Z_i as the set of class labels assigned to the sample i, and $I[]$ a function returning one for a true predicate and zero for a false predicate. Accordingly, we added several metrics for the evaluation of multi-label classifiers only:

Multi-label accuracy[15]: it evaluates also a partial correctness of each assignment. Given N samples, the multi-label accuracy A_{ML} is defined as:

$$A_{ML} = \frac{1}{N}\sum_{i=1}^{N}\frac{|Y_i \cap Z_i|}{|Y_i \cup Z_i|} \tag{4}$$

Relaxed accuracy: it evaluates the proportion of samples for which the original single-label NTP class is assigned. Given N samples, and given NTP_i the original single-label NTP class of sample i, the relaxed accuracy A_{rel} is defined as:

$$A_{rel} = \frac{1}{N}\sum_{i=1}^{N} I[NTP_i \in Z_i] \tag{5}$$

Subset accuracy[15]: it measures the proportion of samples for which all and only the true classes are assigned. Given N samples, the subset accuracy A_{subset} is defined as:

$$A_{subset} = \frac{1}{N}\sum_{i=1}^{N} I[Y_i = Z_i] \tag{6}$$

Average precision: it assesses the correctness of ranking of the assigned classes (it becomes lower when classes to which the sample does not belong are ranked higher than the true classes). Given N samples, the number $|Y_i|$ of true class labels of sample i, and the ranking $r_i(l)$ in Z_i of the class label l in sample i, the average precision AP is defined as:

$$AP = \frac{1}{N}\sum_{i=1}^{N}\frac{1}{|Y_i|}\sum_{l \in Y_i}\frac{|l' \in Y_i : r_i(l') \leq r_i(l)|}{r_i(l)} \tag{7}$$

Hamming loss[15]: it measures, on average, the fraction of class labels incorrectly assigned in each sample; the lower this value, the better the performance. Representing Y_i and Z_i with binary vector representations, the misclassifications can be computed using the X-OR ($\oplus$) binary logic operator. Given $k = 5$ classes, and N samples, the Hamming loss H is defined as:

$$H = \frac{1}{N}\sum_{i=1}^{N}\frac{|Y_i \oplus Z_i|}{k} \tag{8}$$

Training with 10-fold stratified cross-validation and hyper-parameter tuning were applied on both single-label and multi-label classifiers; in order to select the best model of each algorithm, accuracy was used for single-label classifiers, while Hamming loss for multi-label ones.

3.5 Survival analysis and clinical correlates

Kaplan-Meier curves[16] were calculated for each CRIS class, to show the estimated patient survival probability within a fixed number of months. These curves are drawn using survival annotations; specifically, the *disease free months* annotation represents the number of months the patient survived without disease up to the registered event, represented by the *disease free status* annotation. This status can specify that the patient is either disease-free, or that had a recurrence. Censoring occurs for patients whose follow-up has been interrupted after a certain amount of months without disease. The value portrayed in a Kaplan-Meier curve at each month x represents the probability of survival without the disease within the first x months; it is computed as the ratio between the number of patients without disease at month x (disease free) and the patients with active follow up at month x.

In our work, the statistical significance of the association between CRIS class assignments and known sample clinical traits is examined with Fisher tests[17], starting from contingency tables defined as in Table 3 for a generic CRIS class j and a generic clinical trait m.

	Positively annotated for m	Negatively annotated for m
Assigned to class j	a	b
Not assigned to class j	c	d

Table 3. Generic contingency table. a and b represent the number of samples respectively positively and negatively annotated for the feature m that have been assigned to class j. c and d represent the number of samples respectively positively and negatively annotated for the feature m that have not been assigned to class j.

With this contingency table definition, we also computed the *effect size* e_s as in eq. (9), and the *odds ratio*[18] o_r as in eq. (10).

$$e_s = \frac{\frac{a}{a+b}}{\frac{a+c}{a+b+c+d}} \tag{9}$$

$$o_r = \frac{P(m|class = j)/(1 - P(m|class = j))}{P(m|class \neq j)/(1 - P(m|class \neq j))} = \frac{(a/(a+b))/(1-(a/(a+b))}{(c/(c+d))/(1-(c/(c+d))} = \frac{a/b}{c/d} = \frac{a*d}{b*c} \tag{10}$$

The effect size is the ratio between the effective frequency of positively annotated samples in the analyzed class and the expected frequency of positively annotated samples in the entire set; it represents the degree of enrichment, or depletion, when compared with the overall scenario. Conversely, the odds ratio measures the ratio between the proportion of samples positively annotated for m with respect to the samples negatively annotated for m in class j and in class 'not-j'; it represents the degree of association between a class and a property, when compared with that of the complementary classes.

From the effect size formula (eq. (9)), we can notice that it is influenced by the distributions of samples, specifically the overall number of samples and the overall number of samples assigned to class j. Hence, both effect size and odds ratio must be evaluated in our analysis, even more considering that CRIS classes are not balanced. When $e_s > 1$ and $o_r > 1$, the class j is *enriched* with feature m; when instead $e_s < 1$ and $o_r < 1$ the class j is *depleted* from feature m.

3.6 Software

All computations have been performed on RStudio[19] using R 4.0[20]. The libraries used for the computation of results, together with a small description of their use, are here reported: `tidyverse`[21] and `dplyr`[22], for the manipulation of R data frames; `CRISClassifier`[6] for the replication of the original NTP and TSP algorithms; `caret`[8], for the uniformed pipeline for training and testing of single-label approaches; `Kernlab`[10], `e1071`[9], `randomForest`[11], for implementation of Support Vector Machines and Random Forest algorithms; `utiml`[23], for the application of multi-label classifiers and of multi-label specific metrics; `ohenery`[24], for the removal of softmax layer; `ggplot2`[25] and plotly[26] for drawing plots; `survival`[16,27] and `survminer`[28] for the computation of Kaplan-Meier curves.

4 Supplementary results: in-depth analysis of apparent discrepancies between bulk and single-cell class assignments

When comparing multiple subtype assignments of bulk and single cell profiles, for two organoids there was a lack of full coherence. In particular, (i) CRC0515 was univocally assigned to CRIS-E in the bulk data, but single cells were also assigned to other CRIS classes, and (ii) CRC0177 was assigned to CRIS-B as primary class and to CRIS-D as secondary class in the bulk, while a higher number of single cells was assigned to CRIS-D. These two cases are discussed here in more detail.

CRC0515: as shown in main text Figure 2 (reported here in Figure 3 only for cells with unique assignment), cells assigned to non-CRIS-E subtypes have a correlation with the respective centroid lower than CRIS-E cells (see Table 4). Considering the lower number of assigned cells, and the lower correlation with the centroid, it is not unreasonable that the bulk sample reaches significant assignment only to CRIS-E and not to other classes.

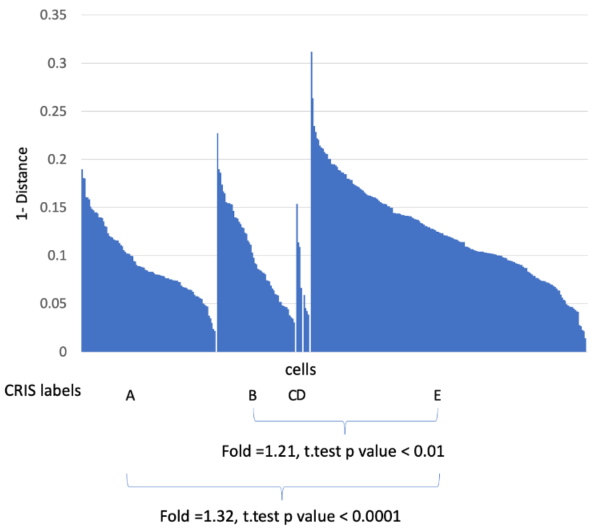

Figure 3. Bar plot representing the proximity to the class centroid of each univocally assigned cell from CRC0515 PDO.

Subtype	Number of uniquely assigned cells	Average correlation
**CRIS-A**	93	0.0932
**CRIS-B**	54	0.1021
**CRIS-C**	4	0.1105
**CRIS-D**	4	0.0462
**CRIS-E**	192	0.1232

Table 4. Number of cells from CRC0515 PDO assigned to each class univocally, together with their average correlation with the class centroid.

CRC0177: while a higher number of cells are assigned to CRIS-D (number of CRIS-B cells = 319; of CRIS-D cells = 406), cells assigned to CRIS-B display higher correlation to the respective centroid (see Figure 4) and therefore a stronger CRIS-B-signature expression (average correlation of CRIS-B cells to CRIS-B centroid = 0.0870; of CRIS-D cells to CRIS-D centroid = 0.0726). It is therefore not unreasonable that the membership to CRIS-B resulted higher in the bulk profile.

References

1. Weinstein, J. N. *et al.* The Cancer Genome Atlas pan-cancer analysis project. *Nat. Genet.* **45**, 1113–1120 (2013).
2. McCarthy, D. J., Chen, Y. & Smyth, G. K. Differential expression analysis of multifactor RNA-seq experiments with respect to biological variation. *Nucleic Acids Res.* **40**, 4288–4297 (2012).
3. Robinson, M. D., McCarthy, D. J. & Smyth, G. K. edgeR: a Bioconductor package for differential expression analysis of digital gene expression data. *Bioinformatics* **26**, 139–140 (2010).

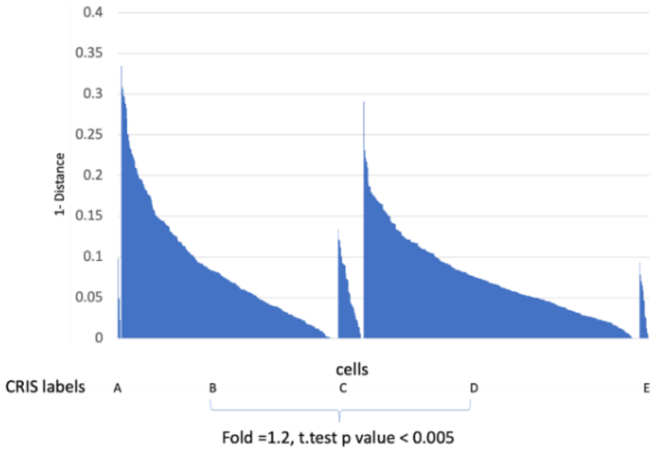

Figure 4. Bar plot representing the proximity to the class centroid of each univocally assigned cell from CRC0177 PDO.

4. Bertotti, A. *et al.* A molecularly annotated platform of patient-derived xenografts ("xenopatients") identifies HER2 as an effective therapeutic target in cetuximab-resistant colorectal cancer. *Cancer Discov.* **1**, 508–523 (2011).
5. Lee, H. O. *et al.* Lineage-dependent gene expression programs influence the immune landscape of colorectal cancer. *Nat. Genet.* **52**, 594–603 (2020).
6. Isella, C. *et al.* Selective analysis of cancer-cell intrinsic transcriptional traits defines novel clinically relevant subtypes of colorectal cancer. *Nat. Commun.* **8**, 15107 (2017).
7. Entrez Gene. https://www.ncbi.nlm.nih.gov/gene/.
8. Kuhn, M. *caret: Classification and Regression Training* (2020). R package version 6.0-86.
9. Meyer, D., Dimitriadou, E., Hornik, K., Weingessel, A. & Leisch, F. *e1071: Misc Functions of the Department of Statistics, Probability Theory Group (Formerly: E1071), TU Wien* (2020). R package version 1.7-4.
10. Karatzoglou, A., Smola, A., Hornik, K. & Zeileis, A. kernlab – an S4 package for kernel methods in R. *J. Stat. Softw.* **11**, 1–20 (2004).
11. Liaw, A. & Wiener, M. Classification and regression by randomForest. *R News* **2**, 18–22 (2002).
12. Chen, T. & Guestrin, C. XGBoost: A scalable tree boosting system. In *Proceedings of the 22nd ACM SIGKDD international conference on knowledge discovery and data mining*, 785–794 (2016).
13. Hand, D. J. & Till, R. J. A simple generalisation of the area under the ROC curve for multiple class classification problems. *Mach. Learn.* **45**, 171–186 (2001).
14. Flach, P. & Kull, M. Precision-recall-gain curves: PR analysis done right. *Adv. Neural Inf. Process. Syst.* **28**, 1–9 (2015).
15. Gibaja, E. & Ventura, S. A tutorial on multi-label learning. *ACM Comput. Surv.* **47**, 52.1–52.38 (2015).
16. Terry M. Therneau & Patricia M. Grambsch. *Modeling Survival Data: Extending the Cox Model* (Springer, New York, 2000).
17. Sprent, P. *Fisher Exact Test* (Springer Berlin Heidelberg, Berlin, Heidelberg, 2011).
18. Agresti, A. *Categorical data analysis*, vol. 482 (John Wiley & Sons, 2003).
19. RStudio Team. *RStudio: Integrated Development Environment for R*. RStudio, PBC., Boston, MA (2020).
20. R Core Team. *R: A Language and Environment for Statistical Computing*. R Foundation for Statistical Computing, Vienna, Austria (2020).
21. Wickham, H. *et al.* Welcome to the tidyverse. *J. Open Source Softw.* **4**, 1686 (2019).
22. Wickham, H., François, R., Henry, L. & Müller, K. *dplyr: A Grammar of Data Manipulation* (2020). R package version 1.0.2.
23. Rivolli, A. *utiml: Utilities for Multi-Label Learning* (2020). R package version 0.1.6.
24. Pav, S. E. *ohenery: Modeling of Ordinal Random Variables via Softmax Regression* (2019). R package version 0.1.1.

25. Wickham, H. *ggplot2: Elegant Graphics for Data Analysis* (Springer-Verlag New York, 2016).

26. Sievert, C. *Interactive Web-Based Data Visualization with R, plotly, and shiny* (Chapman and Hall/CRC, 2020).

27. Therneau, T. M. *A Package for Survival Analysis in R* (2020). R package version 3.1-12.

28. Kassambara, A., Kosinski, M. & Biecek, P. *survminer: Drawing Survival Curves using 'ggplot2'* (2020). R package version 0.4.8.
